# Genotoxic and Anti-Migratory Effects of Camptothecin Combined with Celastrol or Resveratrol in Metastatic and Stem-like Cells of Colon Cancer

**DOI:** 10.3390/cancers16193279

**Published:** 2024-09-26

**Authors:** Helena Moreira, Anna Szyjka, Dorota Bęben, Oliwia Siwiela, Anna Radajewska, Nadia Stankiewicz, Małgorzata Grzesiak, Benita Wiatrak, Fathi Emhemmed, Christian D. Muller, Ewa Barg

**Affiliations:** 1 Department of Basic Medical Sciences, Faculty of Pharmacy, Wroclaw Medical University, 50-556 Wroclaw, Poland; 2 The Hubert Curien pluridisciplinary Institute-IPHC, UMR 7178, University of Strasbourg, 67401 Illkirch, France; 3 Faculty of Pharmacy, Wroclaw Medical University, 50-556 Wroclaw, Poland; 4 Division of Clinical Chemistry and Laboratory Hematology, Department of Medical Laboratory Diagnostics, Faculty of Pharmacy, Wroclaw Medical University, 50-556 Wroclaw, Poland

**Keywords:** metastatic colon cancer, cancer stem cells, camptothecin, celastrol, resveratrol, DNA damage, apoptosis, migration

## Abstract

**Simple Summary:**

Standard chemotherapy is still ineffective in metastatic colon cancer and does not target cancer stem cells (CSCs). The combination of standard cytostatic drugs with natural compounds is considered a promising approach to improving cancer cell sensitivity to treatment. Here, a combination of camptothecin (CPT) with celastrol (CEL) or resveratrol (RSV) as a potential strategy to target metastatic and CSCs of colon cancer was investigated in vitro. γH2AX+ and Fast-Halo assays were used to evaluate genotoxicity, an annexin-V assay was used to rate the level of apoptosis, and a scratch test was used to assess antimigratory capacity. All combinations improved the genotoxicity of CPT, achieving a better efficacy with CPT-CEL combination in LOVO and CPT-RSV in LOVO/DX cells. No improvement in the pro-apoptotic effect of CPT was observed. Furthermore, higher efficiency in inhibiting cancer cell migration was noted.

**Abstract:**

**Background**: Colorectal cancer is one of the leading and most lethal neoplasms. Standard chemotherapy is ineffective, especially in metastatic cancer, and does not target cancer stem cells. A promising approach to improve cancer treatment is the combination therapy of standard cytostatic drugs with natural compounds. Several plant-derived compounds have been proven to possess anticancer properties, including the induction of apoptosis and inhibition of cancer invasion. This study was focused on investigating in vitro the combination of camptothecin (CPT) with celastrol (CEL) or resveratrol (RSV) as a potential strategy to target metastatic (LOVO) and stem-like (LOVO/DX) colon cancer cells. **Methods**: The genotoxic effects that drive cancer cells into death-inducing pathways and the ability to inhibit the migratory properties of cancer cells were evaluated. The γH2AX+ assay and Fast-Halo Assay (FHA) were used to evaluate genotoxic effects, the annexin-V apoptosis assay to rate the level of apoptosis, and the scratch test to assess antimigratory capacity. **Results**: The results showed that both combinations CPT-CEL and CPT-RSV improve general genotoxicity of CPT alone on metastatic cells and CSCs. However, the assessment of specific double-stranded breaks (DSBs) indicated a better efficacy of the CPT-CEL combination on LOVO cells and CPT-RSV in LOVO/DX cells. Interestingly, the combinations CPT-CEL and CPT-RSV did not improve the pro-apoptotic effect of CPT alone, with both LOVO and LOVO/DX cells suggesting activation of different cell death mechanisms. Furthermore, it was found that the combinations of CPT-CEL and CPT-RSV improve the inhibitory effect of camptothecin on cell migration. **Conclusions**: These findings suggest the potential utility of combining camptothecin with celastrol or resveratrol in the treatment of colon cancer, including more aggressive forms of the disease. So far, no studies evaluating the effects of combinations of these compounds have been published in the available medical databases.

## 1. Introduction

Around the world, an increasing number of people are affected by various types of difficult-to-treat as well as lethal forms of tumors, including metastatic cancer. Colorectal cancer (CRC) affects more than 1.85 million people and causes 850,000 deaths each year, third on the list of cancer mortality. In the United States alone, 53,200 deaths were reported in 2020. Overall, 20% of newly diagnosed cases are found to have metastases, and another 25% develop them at a later stage [1]. The incidence of CRC is projected to increase to 3.2 million new cases by 2040 [2]. Diseases and habits of the 21st century such as obesity, a sedentary lifestyle, and smoking contribute to the increase in statistics [3].

The current treatment alternatives for CRC are surgery assisted by systemic therapy, i.e., chemotherapy, radiotherapy, immunotherapy, and targeted and palliative therapy [4,5]. Unfortunately, due to the numerous side effects of chemotherapeutic drugs and the biological characteristics and heterogeneity of cancer cells, the prognosis remains poor, especially in patients with metastatic colorectal cancer (mCRC) with lung or peritoneal metastases [5,6,7]. In addition, patients with distant metastasis or recurrence are usually not candidates for surgical cure [4]. The standard of care for mCRC is based on fluoropyrimidines and irinotecan or oxaliplatin in a doublet or triplet therapy in some patients [5,8]. Chemotherapy combined with a targeted drug (anti-EGFR, anti-VEGF, or TKI) extends the life of patients with a median survival surpassing 2 years. Regrettably, chemotherapy combined with two targeted drugs increases intolerable toxicity. Recently, immune checkpoint blockade (ICB) therapy has contributed to significant progress in treating advanced malignancies. Patients who benefit from ICB can experience long-term health improvements. However, this therapy also has limitations, i.e., it is beneficial for mCRC patients with microsatellite instability (MSI-H) at the early stages of the disease, but patients who initially respond to treatment may later develop acquired resistance [9].

Despite significant progress in mCRC therapeutic approaches, none are curative in most advanced cancer cases. The majority of patients fail or relapse regardless of initially successful therapy, which is associated with the development of multidrug resistance (MDR) during or after treatment [9]. Currently, MDR is attributed to cancer stem cells (CSCs), referred to as colon cancer stem cells for CRC. CSCs are responsible for therapy resistance, progression to aggressive form, and metastasis development. In addition, the presence of CSCs manages the persistence of the so-called minimal residual disease (MRD) and cancer recurrence [7]. Therapy targeting CSCs currently seems to be the most promising for the complete eradication of the disease. However, clinical trials of anti-CSC therapy are rare. So far, a few pharmacological agents have been studied: napabucasin, OMP-21M18, and mithramycin, from which only napabucasin demonstrates relevant results in phase I/II development and has entered phase III [7]. Therefore, the search for new alternative mCRC treatments that will be effective against CSCs is still necessary.

In recent years, there has been an increasing interest in natural compounds as potential adjuvant therapeutics in the treatment of various cancers [10,11]. Due to their pleiotropic effect, i.e., the ability to interfere with various intracellular pathways in cancer cells, they can be more effective in eliminating therapy-resistant aggressive cancer cells, including CSCs. Several studies have shown the efficacy of curcuminoids, terpenoids, isothiocyanates, alkaloids, and isoflavones in targeting stem cell and metastatic biomarkers in CSCs [12]. In addition, natural compounds are potentially safe for healthy cells and should reduce side effects. Therefore, combining natural compounds with chemotherapeutic drugs might provide an ideal therapeutic strategy that targets both rapidly proliferating cancer cells of the tumor bulk and resistance to treatment cancer stem cells.

Celastrol (CEL) and resveratrol (RSV) are natural compounds with therapeutic effects against various ailments, as well as chemopreventive and anticancer properties. CEL is a terpene isolated from extracts of the roots of *Tripterygium wilfordii* [13]. RSV, a phytoalexin antioxidant, belongs to a class of polyphenolic compounds named stilbenes, found in red grapes [14]. Both compounds have been noted to reduce various human cancers, including blood, breast, esophagus, stomach, colon, lung, liver, kidney, bladder, prostate, ovary, cervix, thyroid, brain, head and neck, eye, skin, and bone. In addition, CEL and RSV have been shown to have the ability to interact with various cellular targets, which allows them to be particularly promising phytochemicals for colon cancer therapy [14,15]. Camptothecin (CPT) is a quinoline-type alkaloid isolated from the Chinese tree’s cortex *Camptotheca acuminate* [16]. CPT has been shown to inhibit cancer cell proliferation by blocking DNA topoisomerase 1, the enzyme involved in the replication and repair of the DNA. Inhibition of topoisomerase 1 activity results in the accumulation of the DNA fragments and activation of cell death pathways, which ultimately leads to cancer cell death [17]. Due to the increased resistance of cancer cells to CPT treatment and its toxicity, several CPT analogs have been developed so far, including irinotecan (IRY) [18]. IRY is currently registered in oncology for treating metastatic or advanced solid tumors, such as colon, gastric, or pancreatic cancer [19]. However, de novo or acquired clinical resistance to IRY and other camptothecins derivatives is still common. Therefore, there is still a need for new approaches, e.g., pharmacological modulation to circumvent resistance mechanisms, increase antitumor activity, and reduce systemic toxicity [20].

The still-current problem of metastatic cancer treatment related to the presence of CSCs and the development of drug resistance prompted us to look for a new strategy that could be useful in colorectal cancer. Previously, we have shown that CEL and RSV used in monotherapy exert an anticancer activity against metastatic colon cancer cells and CSC-like cells through various mechanisms [21]. Thus, both compounds have very promising properties that could improve the effectiveness of a classic cytotoxic drug. In this study, we focused on evaluating the usefulness of combined therapy of CPT with CEL or RSV as a potential therapeutic strategy to eliminate metastatic cells and CSCs of colon cancer. These compound combinations have not yet been studied, especially in the context of comparing effects on metastatic cancer and CSCs. Here, we investigated the genotoxic effects that drive cancer cells into apoptotic or necrotic death pathways, as well as the ability of such a therapy to inhibit the migratory properties of cancer cells. The study was conducted using a LOVO cell line established from metastatic nodules from a colon adenocarcinoma cell line that is Dukes’type C, from a grade IV patient, enriched in CSCs, doxorubicin-resistant, and LOVO/DX. Our research shows that these compounds can significantly improve the genotoxic and anti-migratory effects of CPT, opening up new possibilities for more effective anti-cancer therapies.

## 2. Materials and Methods

### 2.1. Materials

Lonza (Basel, Switzerland) supplied the following supplies: DMEM/F12 (Dulbecco’s Modified Eagle’s Medium: Nutrient Mixture F-12), HBSS (Hank’s Balanced Salt Solution), DPBS (Dulbecco’s Phosphate-Buffered Saline), FBS (fetal bovine serum), ultra glutamine 1, and gentamicin sulfate. Gibco was the source of TrypLETM Express (Waltham, MA, USA). Paraformaldehyde (PFA), DMSO (Dimethyl Sulfoxide), camptothecin (97% of purity), resveratrol (99% of purity), DAPI (4′,6-Diamidino-2-phenylindole dihydrochloride), Agarose Type I-A (LMP—low melting point), and Agarose Type II-A (NMP—normal melting point) were purchased from Sigma Aldrich (St. Louis, MO, USA). MSC attachment solution was from Sartorius Poland (Kostrzyn, Poland). Celastrol (98% of purity) was purchased from Cayman Chemical (Ann Arbor, MI, USA). Ethanol 96% was from Chempur (Piekary Śląskie, Poland). FcR blocking reagents were from Miltenyi Biotec (Auburn, CA, USA). Phospho-histone H2A.X (Ser139) monoclonal antibody (CR55T33) Alexa Fluor 488 eBioscience^TM^ was obtained from Invitrogen (Carlsbad, CA, USA). Alexa Fluor^®^488 AnnexinV/Dead Cell Apoptosis Kit was purchased from Life Technologies (Warsaw, Poland). Incucyte^®^ Imagelock 96-Well Plates (Sartorius) were from OMIXYS (Warsaw, Poland).

### 2.2. Drug Solutions

Stock solutions of celastrol and resveratrol were prepared by dissolving the compounds in DMSO to a concentration of 10 mM and camptothecin to a concentration of 1mM. The stock solutions were stored at −20 °C. Before each experiment, the working solutions were prepared by dilution of stock solutions in a culture medium. The tested concentrations of the drugs were 5, 10, 20, and 40 µM for camptothecin, 1.25, 2.5, 5, and 10 µM for celastrol, and 2.5, 5, 10, and 20 for resveratrol, with the following combinations: CPT20 µM/CEL1.25 µM, CPT20 µM/CEL2.5 µM, CPT20 µM/CEL5 µM, CPT20 µM/CEL10 µM, CPT20 µM/RSV2.5 µM, CPT20 µM/RSV5 µM, CPT20 µM/RSV10 µM, and CPT20 µM/RSV20 µM. The final DMSO concentration in the cell culture did not exceed 0.4% at the highest concentration of the compounds.

### 2.3. Cell Line and Culture Conditions

The LOVO cell line (colon adenocarcinoma cell line, Dukes’type C, grade IV, ATCC^®^ CCL-229^TM^) was obtained from ATCC (American Type Culture Collection, Manassas, VA, USA). LOVO/DX, a doxorubicin-resistant cell line enriched in CSCs, was derived from the original LOVO cells by continuous in vitro exposure to low doses of doxorubicin for 3 months [22]. The characteristics of CSCs in the LOVO/DX cell line are described in our previous paper [23]. The cells were cultured in DMEM/F12 medium, supplemented with 10% FBS, 2 mM L-glutamine, and 25 µg/mL gentamicin at 37 °C in a CO_2_-incubator. The cells were subcultured twice a week using the TrypLETM Express solution.

### 2.4. Apoptosis and Necrosis Detection

For discrimination between viable, apoptotic, and necrotic cells, the Alexa Fluor^®^488 AnnexinV/Dead Cell Apoptosis Kit (Invitrogen/Molecular Probes) was used. The kit contains recombinant annexin V conjugated to the Alexa Fluor^®^488 dye for staining apoptotic cells and Propidium Iodide (PI) for staining dead cells.

Before staining, cells (7.5 × 10^5^) were incubated with tested compounds or their combination for 24 h (37 °C, 5% CO_2_) in a 6-well plate (TPP, Trasadingen, Switzerland). Following incubation, cells were detached using Accutase^TM^ Cell Detachment (BD Biosciences, Franklin Lakes, NJ, USA) and washed with cold HBSS. The cell samples were resuspended in 100 μL of ice-cold 1x binding buffer (BB). Cells were stained with 5 µL of FITC Annexin V Alexa Fluor^®^488 dye and 1 µL of PI for 15 min in the dark at room temperature according to the manufacturer’s recommendations. Samples were immediately analyzed with a CyFlow^®^SPACE flow cytometer (Sysmex-Partec, Görlitz, Germany) using 488 nm laser excitation with a 536/40 (BP) filter for Annexin V-FITC and 675/20 (BP) for PI. Granulation, size, and fluorescence intensity were recorded for at least 10,000 cells. The results were then displayed in a dot-plot format. Four populations of the cells were distinguished, as follows:

Non-apoptotic, viable cells: Alexa Fluor^®^488 AnnexinV (−) and PI (−)

Early apoptotic cells: Alexa Fluor^®^488 AnnexinV (+) and PI (−)

Late apoptotic cells: Alexa Fluor^®^488 AnnexinV (+) and PI (+)

Necrotic cells: Alexa Fluor^®^488 AnnexinV (−) and PI (+)

The samples were analyzed using the FloMAx Software 2011 v2.81 (Partec GmbH, Münster, Germany). The percentage of cells in each population was computed.

### 2.5. γH2AX and γH2AX Cell Cycle Detection

γH2AX, the phosphorylated form of H2AX, and cell cycle were detected using double staining with phospho-histone H2A.X (Ser139) monoclonal antibody Alexa Fluor 488 and DAPI, as previously described [21,24]. Briefly, cells (7.5 × 10^5^) were incubated with tested compounds or their combination for 24 h (37 °C, 5% CO_2_) in a 6-well plate. Then, the cells were extracted with TrypLETM Express solution, underwent a two-fold cleaning procedure with cold HBSS, and were fixed for ten minutes on ice with 2% paraformaldehyde (PFA). The cells were permeabilized with ice-cold 70% ethanol and maintained in this solution at −20 °C for a period of five to seven days, following two washing procedures with cold HBSS/1% BSA. Prior to antibody staining, the cells underwent two rounds of washing with HBSS/1% BSA. Following resuspension of the cell pellets in 100 μL of HBSS/1% BSA, containing 2 μL of phospho-histone H2A.X (Ser139) monoclonal antibody Alexa Fluor 488 and incubated for 60 min in the dark. After one washing step with HBSS/1% BSA, the cells were resuspended in 500 µL of HBSS/1% BSA and stained with 1 µL of DAPI solution (1 mg/mL) for 15 min. Then, the samples were acquired on a CyFlow^®^SPACE flow cytometer (Sysmex-Partec, Görlitz, Germany). A 488nm laser excitation and 536/40 (BP) filter were used for FITC fluorescence detection. A 375nm laser excitation and 455/50 nm (BP) filter were used for DAPI fluorescence detection. A total of 10,000–20,000 cells per sample were acquired. The results were analyzed with FCS Express 4 flow software (De Novo Software, Glendale, CA, USA).

### 2.6. Fast Halo Assay (FHA)

The fast halo assay (FHA) enables rapid testing of genotoxicity by evaluating DNA diffusion. Cells (7.5 × 10^5^) were seeded in 6-well plates and incubated with tested compounds or their combination for 24 h (37 °C, 5% CO_2_). Then, cells were detached with TrypLE^TM^ Express solution and washed with DPBS. After the washing step, the cells were resuspended in 30 μL DPBS and placed in a water bath at 37 °C, and 120 μL of 1.2% LMP (low melting point) agarose was added to the cell suspension. Then, 80 μL of cell suspension in LMP agarose was added to the slide coated with NMP (normal melting point) agarose and allowed to gel for 10 min. The slides were placed in the lysis buffer overnight at 4 °C. Slides were drained and placed in an alkaline buffer (pH = 13) for 30 min, and then soaked twice for 5 min in the neutralizing buffer. Slides were stained with DAPI solution [20 µg/mL] for 20 min and photographed under a fluorescence microscope. Using the proprietary Halo Assay software (by Benita Wiatrak [24]), the size of the fluorescent glow around the cell nucleus was assessed. This glow is formulated by relaxed chromatin. The program calculates the ratio of the diameter of the cell nucleus to the diameter of the HALO glow (Figure S3).

### 2.7. Scratch Assay

To determine the effect of the tested compounds on cell migration, a scratch assay (wound healing assay) was performed. Briefly, cells were seeded at a density of 35,000 cells/200 µL per well in Incucyte^®^ Imagelock 96-Well Plates coated with fibronectin (MSC attachment solution diluted 1:100 using sterile DPBS). Cells were allowed to attach and grow to full confluency for 24 h. A scratch was created in the center of each well using an Incucyte^®^ 96-Well Woundmaker Tool (Sartorius Poland, Kostrzyn, Poland), and the wells were washed twice with HBSS to remove detached cells and debris. Solutions of the tested compounds or their combination prepared in fresh culture medium were added to the wells, and the plates were incubated in the Incucyte^®^ S3 incubator for 72 h (37 °C/5% CO_2_) [25]. Images of the scratch were taken using an Incucyte^®^ Live-Cell Analysis microscope (Sartorius Poland, Kostrzyn, Poland) at 24-h intervals. The images were analyzed using IncuCyte S3 Software (Incucyte^®^ Scratch Wound Analysis Software Module), an image analysis software, to measure the relative wound density (RWD) at various time points. The RWD over time for each well was plotted to determine the rate of scratch closure.

### 2.8. Statistical Analysis

The statistical significance of the results was calculated using Statistica v13.3 software. All figures were carried out with GraphPad Prism 7.02 for Windows (GraphPad Software, San Diego, CA, USA). The data had a normal distribution and equality of variance; therefore, one-way ANOVA and Tukey’s post-hoc analysis were performed. The significance point was *p* ≤ 0.05.

## 3. Results

### 3.1. Evaluation of Genotoxic Effects

#### 3.1.1. Induction of Double-Stranded DNA Breaks (DSBs)

**Figure 1:**
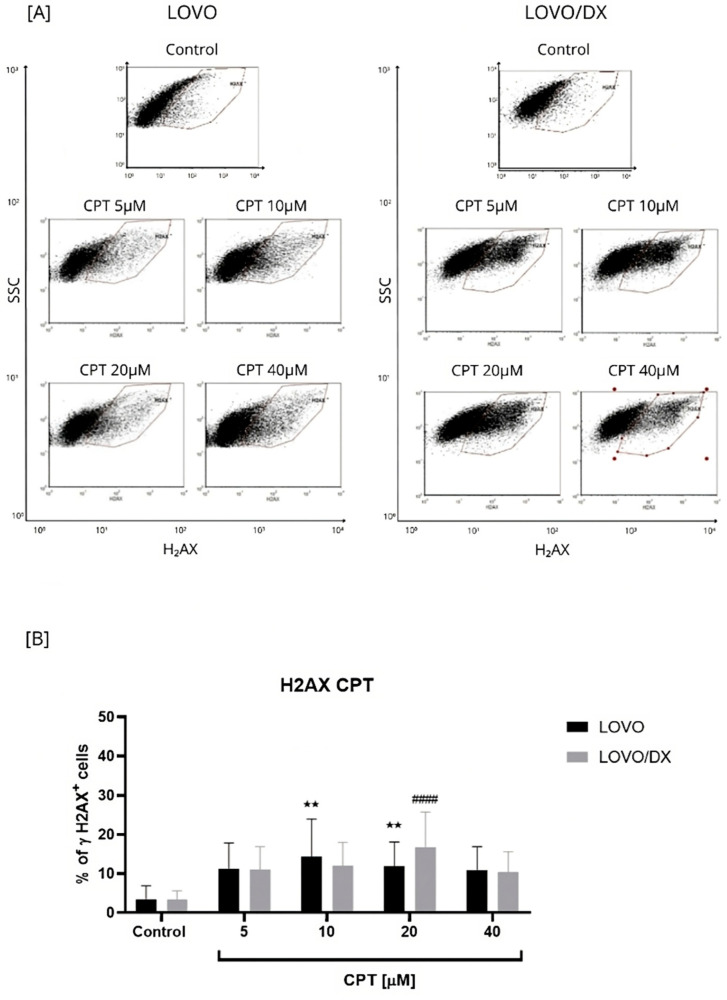
Effects of camptothecin (CPT) on the frequency of γH2AX+ cells in LOVO and LOVO/DX (**B**) cells. Column bar graph showing the mean ± SD of five independent experiments. ** *p* < 0.01 for LOVO, control vs. CPT, ^####^ *p* < 0.0001 for LOVO/DX, control vs. CPT. (**A**) Representative cytograms of flow cytometric analysis. Control: cells incubated in the presence of the solvent (DMSO).

**Figure 2:**
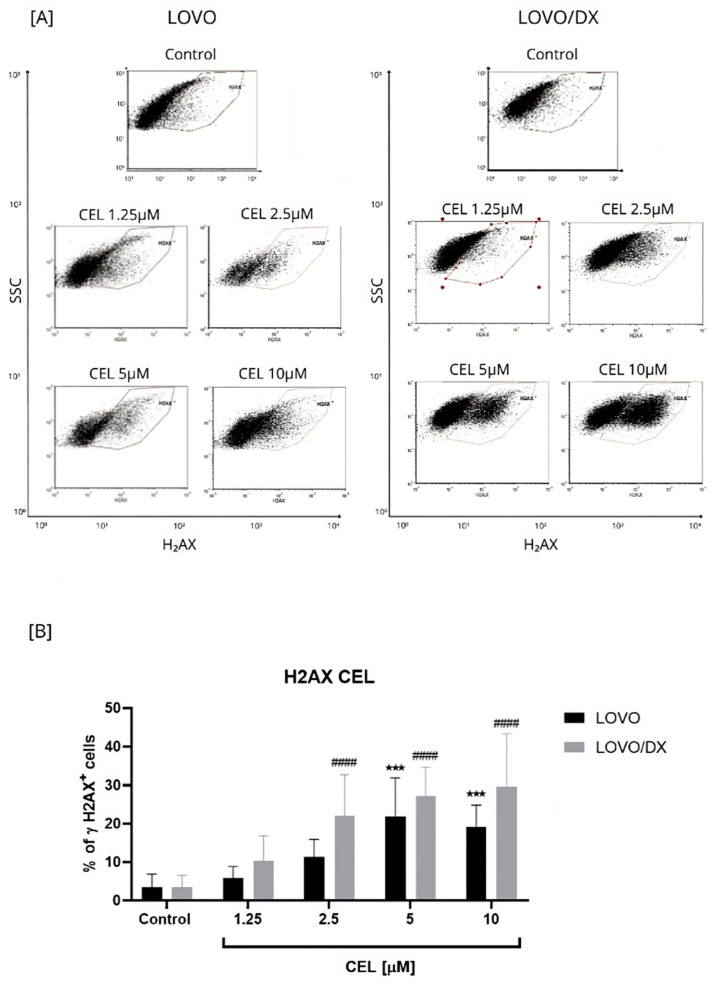
Effect of celastrol (CEL) on the frequency of γH2AX+ cells in LOVO and LOVO/DX cells (**B**). Column bar graph showing the mean ± SD of five independent experiments. *** *p* < 0.001 for LOVO, control vs. CEL, ^####^ *p* < 0.0001 for LOVO/DX, control vs. CEL. (**A**) Representative cytograms of flow cytometric analysis. Control: cells incubated in the presence of the solvent (DMSO).

**Figure 3:**
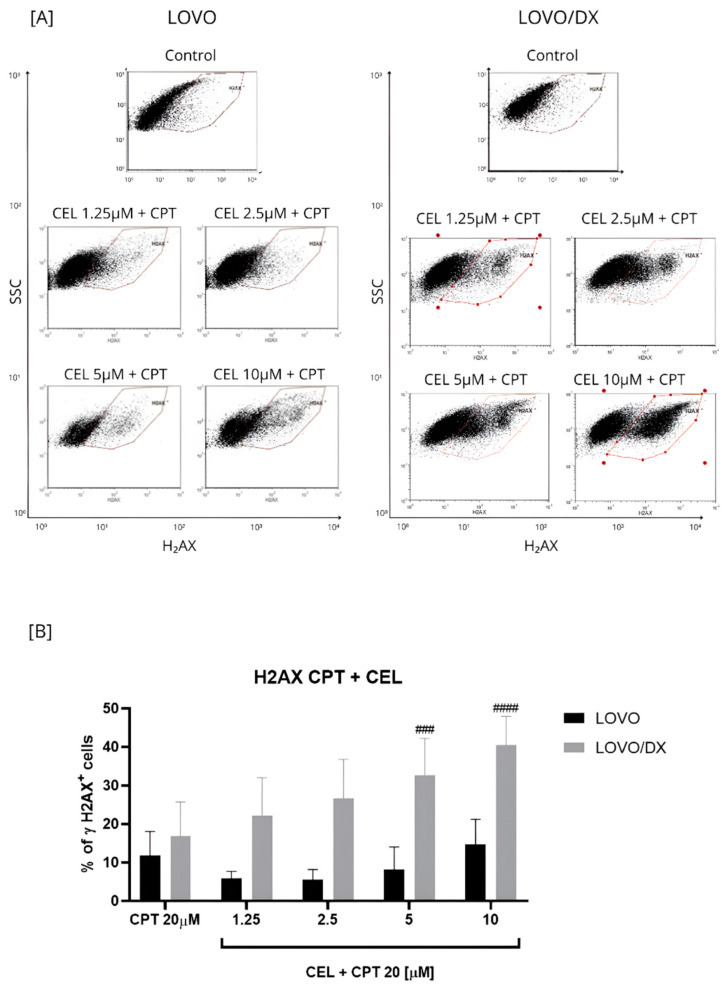
Effects of combinations of camptothecin (CPT, 20 µM) with celastrol (CEL) on the frequency of γH2AX+ cells in LOVO and LOVO/DX (**B**) cells. Column bar graph showing the mean ± SD of five independent experiments. ^###^ *p* < 0.001, ^####^ *p* < 0.0001 for LOVO/DX, CPT vs. CEL + CPT 20. (**A**) Representative cytograms of flow cytometric analysis.

**Figure 4:**
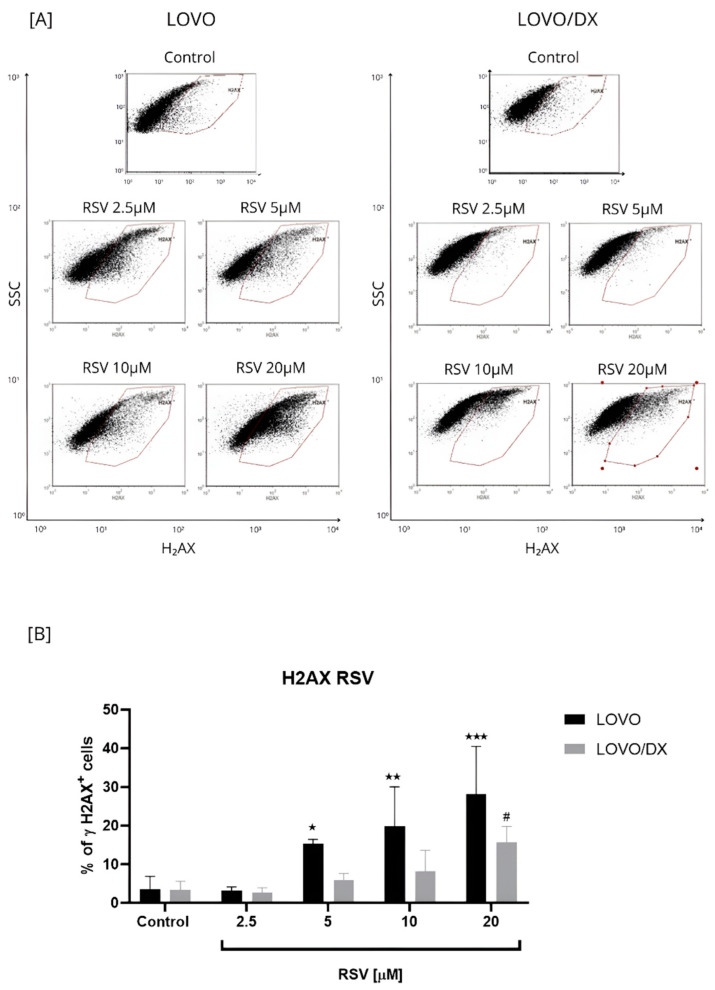
Effects of resveratrol (RSV) on the frequency of γH2AX+ cells in LOVO and LOVO/DX (**B**) cells. Column bar graph showing the mean ± SD of five independent experiments. * *p* ≤ 0.05, ** *p* < 0.01, *** *p* < 0.001 for LOVO, control vs. RSV and ^#^ *p* ≤ 0.05 for LOVO/DX, control vs. RSV. (**A**) Representative cytograms of flow cytometric analysis.

**Figure 5:**
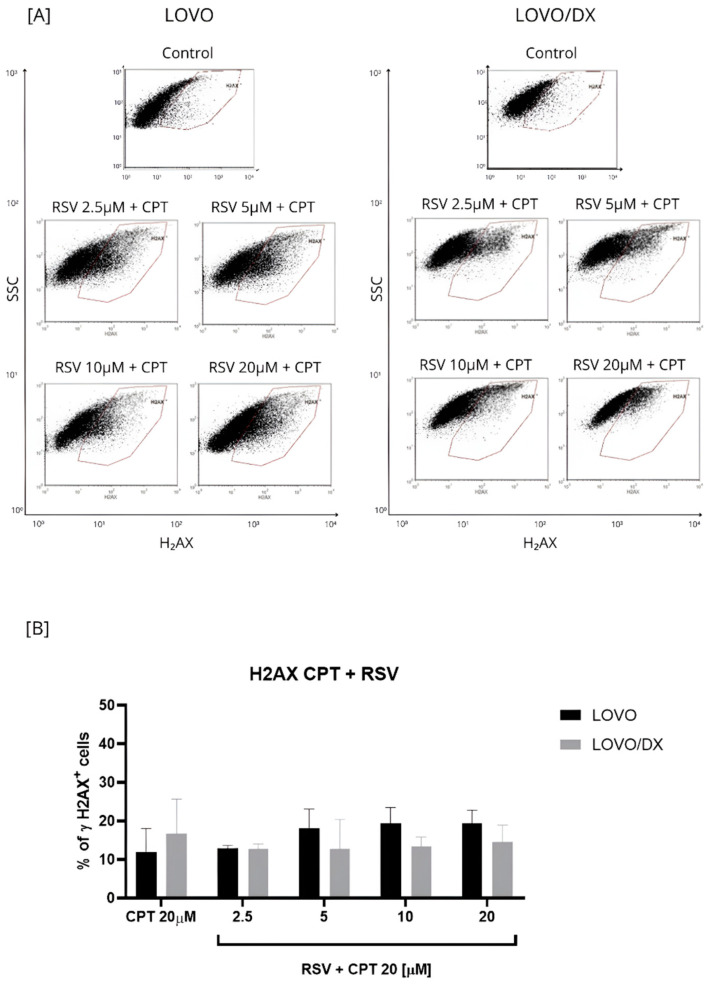
Effects of resveratrol (RSV) and its combinations with camptothecin (CPT) on the frequency of γH2AX+ cells in LOVO and LOVO/DX (**B**) cells. Column bar graph showing the mean ± SD of five independent experiments. *p* ≤ 0.05. (**A**) Representative cytograms of flow cytometric analysis.

First, we evaluated the potential of tested compounds and their combination to induce double-stranded DNA breaks (DSBs) in colorectal cancer cells. DSBs are among the most dangerous forms of DNA damage that lead to cell death, most often by apoptosis. The appearance of these DNA damages in the cell nucleus is associated with the phosphorylation of histone H2AX at position Ser139. The phosphorylated form of H2AX is referred to as γH2AX. Since the number of γH2AX foci in the nucleus is directly proportional to the number of DSBs formed, γH2AX formation has been considered a sensitive marker of double-stranded DNA breaks and is an early indicator of cancer cell sensitivity to chemotherapy [24,26]. Analysis of γ-H2AX by microscopy (immunocytochemical detection) is considered to be the most sensitive and specific detection method of DSB and is a gold standard for single DSB analysis [27]. Other techniques like immunoblotting, and enzyme-linked immunosorbent assay (ELISA), and flow cytometry are also useful [28]. A flow cytometry-based γ-H2AX assay has been reported to be sensitive and precise in measuring DNA damage [29]. In this study, the immunofluorescent γH2AX labeling with the monoclonal antibody anti-phospho-histone γH2AX (Ser139) Alexa Fluor 488 was used and the percentage of γH2AX+ cells was assessed by flow cytometry. The representative cytograms of flow cytometric analysis are presented in Figure 1, Figure 2, Figure 3, Figure 4 and Figure 5.

##### Camptothecin-Induced γH2AX Formation

As seen in Figure 1, camptothecin induces the formation of γH2AX in both LOVO and LOVO/DX cells to a similar extent, regardless of the concentration used. The number of detected γH2AX+ cells increased up to 14% in LOVO and 17% in LOVO/DX cells. In CSC-like colon cancer cells (LOVO/DX cells), CPT, at a concentration of 20 µM, was the most effective in inducing harmful DSBs. Therefore, this concentration of CPT was selected for further studies on the effectiveness of the combination of CEL or RSV with CPT.

##### Induction of γH2AX by the Combination of Camptothecin with Celastrol

Celastrol was more potent in inducing DSBs compared to CPT; up to 22% LOVO and 30% LOVO/DX γH2AX+ cells were detected (Figure 2). In addition, the effect of celastrol on the frequency of γH2AX+ cells was concentration-dependent relative to the control.

The effect of the combination of different CEL concentrations with CPT at 20 µM on γH2AX+ formation was compared to the effect of CPT alone (Figure 3 and Figure S1). The CPT-CEL treatment improved the efficiency of CPT (20 µM) in inducing DSB only in LOVO/DX cells. The frequency of detected γH2AX+ cells increased by 3.5–24% over the CPT effect. A statistically significant enhancement was noted for the combination of CPT with CEL at 5 µM and 10 µM. In addition, the combinations of CPT–CEL caused an increase in γH2AX+ formation compared to CEL alone in LOVO/DX cells. In metastatic LOVO cells, the combination of 20 µM CPT with 10 µM CEL resulted in a very slight increase in γH2AX+ formation by about 3%, and it was not statistically significant. Lower celastrol concentrations attenuated the effect of CPT. In addition, the CPT-CEL treatment lowered the frequency of γH2AX+ cells compared to the effect of CEL alone.

##### Induction of γH2AX by the Combination of Camptothecin with Resveratrol

Resveratrol increased the frequency of γH2AX formation in a concentration-dependent manner in LOVO and LOVO/DX cells (Figure 4). However, the percentage of detected γH2AX+ cells was much higher in metastatic disease than in CSC-like colon cancer cells and increased to 28% and 14% for 20 µM RSV in LOVO and LOVO/DX cells, respectively.

The effect of the combination of different RSV concentrations with CPT at 20 µM on γH2AX+ formation was compared to the effect of CPT alone (Figure 5 and Figure S2). The CPT-RSV treatment improves the efficiency of CPT alone in inducing DSB in LOVO cells only. The percentage of detected γH2AX+ cells increased by 6–8% for combinations of 20 µM CPT and 5 µM RSV, 20 µM CPT and 10 µM RSV, and 20 µM CPT and 20 µM RSV, and the effect was statistically significant. However, only the combinations of 20 µM CPT and 2.5 µM RSV and 20 µM CPT and 5 µM RSV increased the frequency of γH2AX+ cells compared to the effect of RSV alone. In LOVO/DX cells, RSV did not enhance the effect of CPT alone on γH2AX+ formation. Nevertheless, the combinations: 20 µM CPT and 2.5 µM RSV, 20 µM CPT and 5 µM RSV, 20 µM CPT and 10 µM RSV caused an increase in γH2AX+ formation compared to RSV alone.

#### 3.1.2. Induction of DNA Fragmentation

The degree of DNA fragmentation as a result of exposure of LOVO and LOVO/DX cells to the compounds was assessed using the FHA (Fast Halo Assay). The FHA test is based on the assessment of DNA diffusion by measuring the size of the glow around the cell nucleus, at the single-cell level, which is created by relaxed chromatin [30] (Figure S3).

**Figure 6:**
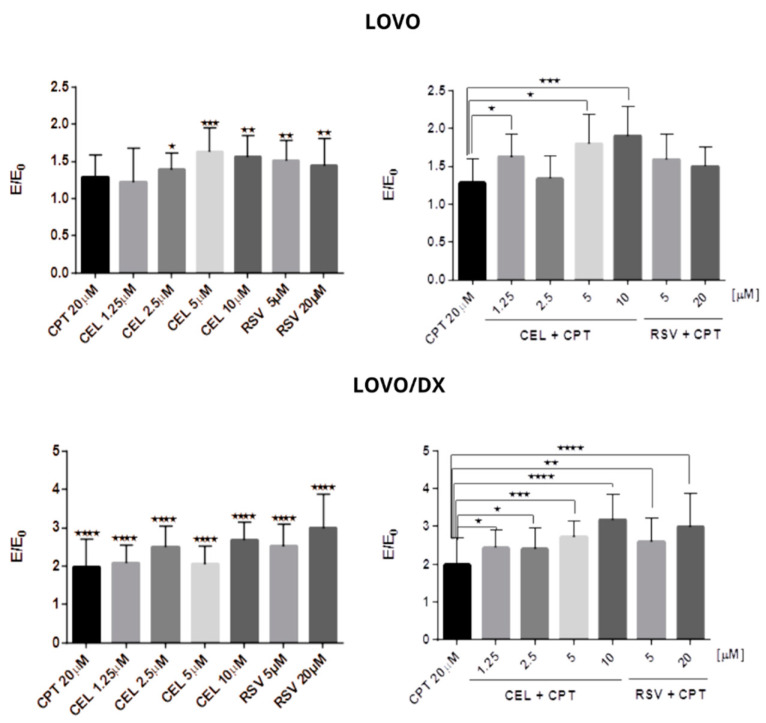
Genotoxic effect of camptothecin (CPT), celastrol (CEL), resveratrol (RSV), and their combinations in LOVO and LOVO/DX cells. The results are expressed as the E/E0 ratio, where E is the mean of the genotoxic effects (measured by Fast Halo assay) in the cells incubated with tested compounds or their combinations and E0 is the mean of the genotoxic effects in the control samples. Control consisted of cells incubated in the presence of the solvent (DMSO). The results are the mean ± SD of five independent experiments. * *p* ≤ 0.05, ** *p* < 0.01, *** *p* < 0.001, **** *p* < 0.0001.

**Figure 7:**
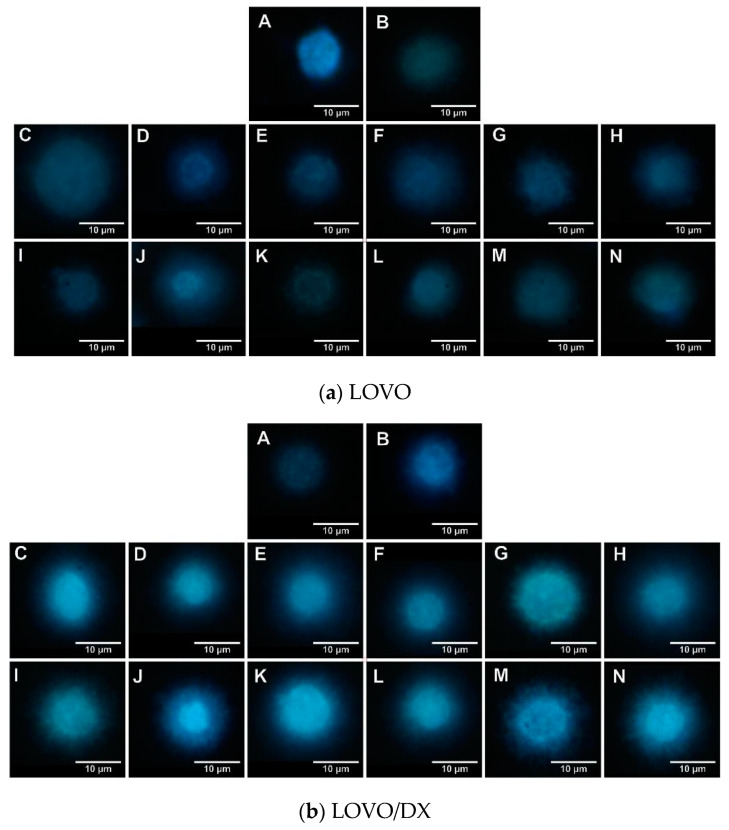
Genotoxic effect of camptothecin (CPT), celastrol (CEL), resveratrol (RSV), and their combinations. Representative photographs of the LOVO (**a**) and LOVO/DX (**b**) cells with a glow around the nucleus in Fast Halo Assay (FHA) are shown. A—control cells, B—CPT 20 µM, C—CEL 1.25 µM, D—CEL 1.25 µM + CPT 20 µM, E—CEL 2.5 µM, F—CEL 2.5 µM + CPT 20 µM, G—CEL 5 µM, H—CEL 5 µM + CPT 20 µM, I—CEL 10 µM, J—CEL 10 µM + CPT 20 µM, K—RSV 5 µM, L—RSV 5 µM + CPT 20 µM, M—RSV 20 µM, N—RSV 20 µM + CPT 20 µM.

Camptothecin, celastrol, and resveratrol strongly induce DNA fragmentation after 24 h of incubation with LOVO and LOVO/DX cells (Figure 6, Figure 7 and Figures S4 and S5). However, a significantly stronger genotoxic effect was observed in LOVO/DX cells for cleastrol in concentrations 2.5 µM and 10 µM and resveratrol at a concentration of 20 µM (mean value: 1.390 vs. 2.501 E/E0, *p* = 0.0309; 1.560 vs. 2.682 E/E0, *p* = 0.0239; 1.440 vs. 2.991 E/E0, *p* = 0.0473 for LOVO vs. LOVO/DX, respectively). There was an increase in the percentage of cells with DNA damage to 99% for CPT, 168% for CEL, and 175% for RSV relative to the control level.

A combination of CPT (20 µM) with CEL or RSV improves the effect of CPT in both cell lines. In LOVO cells, the combination of CPT-CEL caused up to a 60% increase in genotoxic effects compared to CPT alone, and CPT-RSV up to 29%. In LOVO/DX cells, the combinations of CPT-CEL and CPT-RSV induced stronger genotoxic effects, i.e., up to 118% and up to 100% increases in DNA fragmentation were noted, respectively, compared to CPT alone. In addition, the CPT-CEL combination also resulted in an increase in DNA fragmentation compared to CEL alone, while the CPT-RSV combination showed only a slight increase compared to RSV alone in both LOVO and LOVO/DX cells.

#### 3.1.3. Calculation of the Combination Index (CI)

To evaluate the synergistic, additive, or antagonist effect of the combination of camptothecin with celastrol or camptothecin with resveratrol, a combination index (CI) was calculated using CompuSyn software v1.0 (ComboSyn, Inc., Paramus, NJ, USA). The formula used by the software is given in the supplementary materials. The combination index generally defines synergism when CI < 1, the additive effect when CI = 1, and antagonic effects when CI > 1. CI was generated based on the genotoxic effects (γ-H2AX results). As can be seen in Table 1, all combinations of CPT with CEL have synergistic effects on genotoxicity in LOVO/DX cells. Based on the detailed criteria shown in Table S1 [25], the combination of CPT 20 µM + CEL 1.25 µM exhibits a strong synergistic effect. The same compound combinations demonstrated antagonistic effects in LOVO cells. The combination of CPT 20 µM with RSV at 2.5, 5, and 10 µM also exhibits synergistic effects in LOVO/DX (Table 2). Antagonistic interactions were detected for the combination of 20 µM CPT with 20 µM RSV in LOVO/DX cells and for all CPT-RSV combinations in LOVO cells.

### 3.2. Effects of Camptothecin, Celastrol, and Resveratrol and Their Combination on Apoptotic and Necrotic Cell Death

As DNA damage is associated with the initiation of apoptosis, we further evaluated the effect of tested compounds on the frequency of apoptotic cells after 24 h of incubation with LOVO and LOVO/DX cells. Apoptotic cells were detected by flow cytometry using double staining with annexin V-Alexa Fluor 488 and Propidium Iodide (PI). This staining allows the detection of apoptotic (annexin V-Alexa Fluor 488+ cells) and necrotic cells (PI+ cells). The representative cytograms of control LOVO and LOVO/DX cells are presented in Figure S6 (Supplementary Materials).

**Figure 8:**
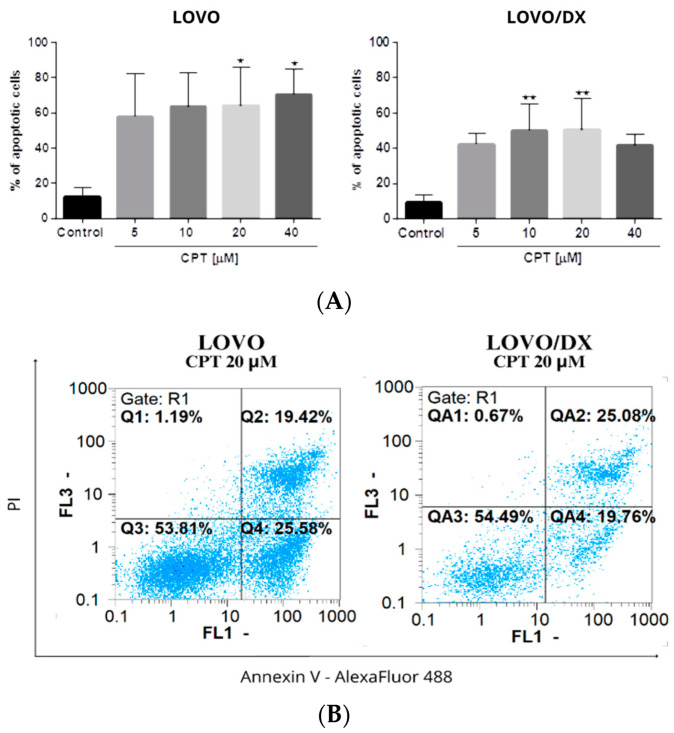
(**A**) The effect of camptothecin (CPT) on the frequency of apoptosis in LOVO and LOVO/DX cells. The results are presented as the mean ± SD of five independent experiments. * *p* ≤ 0.05, ** *p* < 0.01. (**B**) Representative cytograms; FL1- Alexa Fluor^®^488 AnnexinV, FL3-Propidium Iodide (PI).

**Figure 9:**
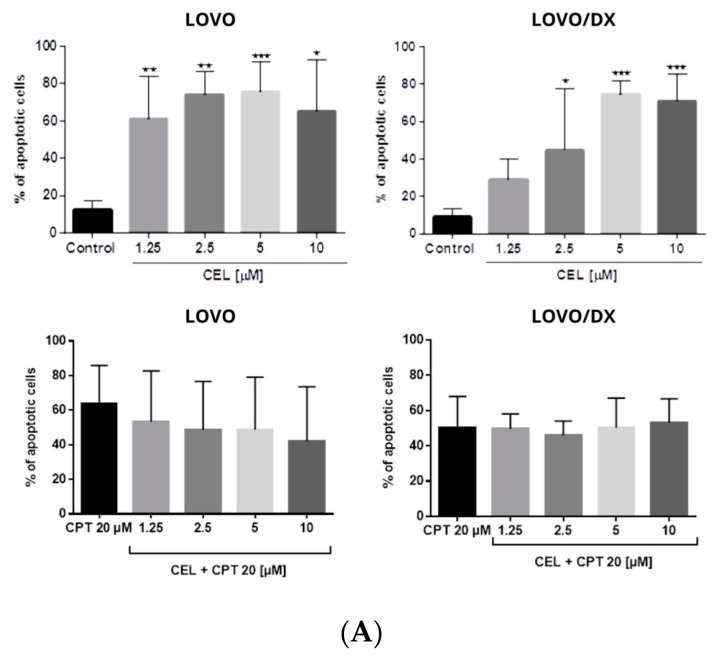

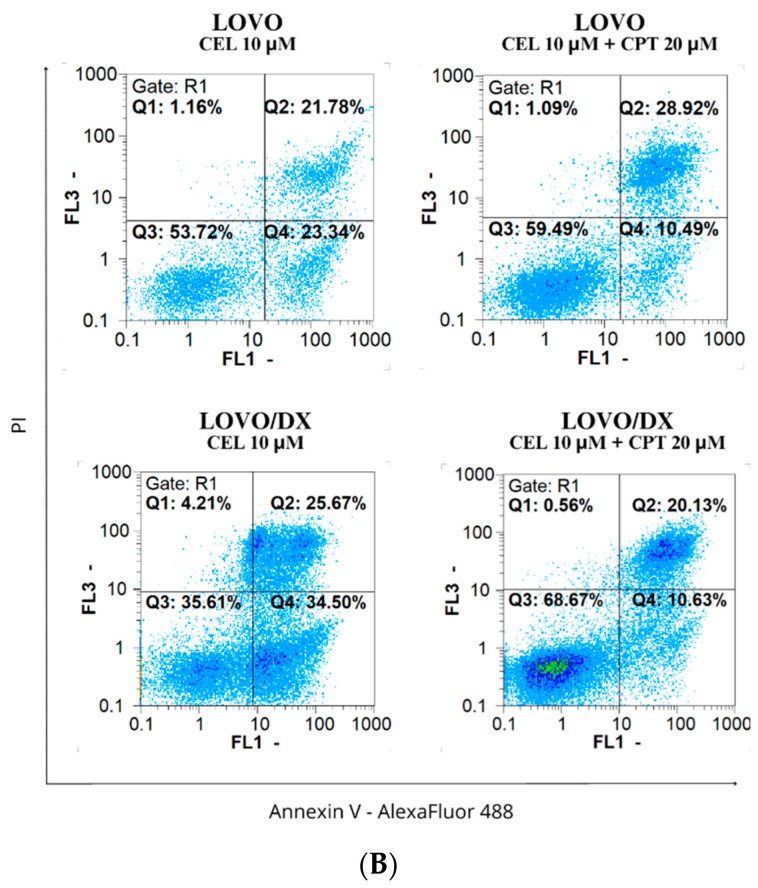
(**A**) Effects of celastrol (CEL) and its combinations with camptothecin (CEL) on the frequency of apoptosis in LOVO and LOVO/DX cells. The results are presented as the mean ± SD of five independent experiments. * *p* ≤ 0.05, ** *p* < 0.01, *** *p* < 0.001. (**B**) Representative cytograms; FL1- Alexa Fluor^®^488 AnnexinV, FL3-Propidium Iodide (PI).

**Figure 10:**
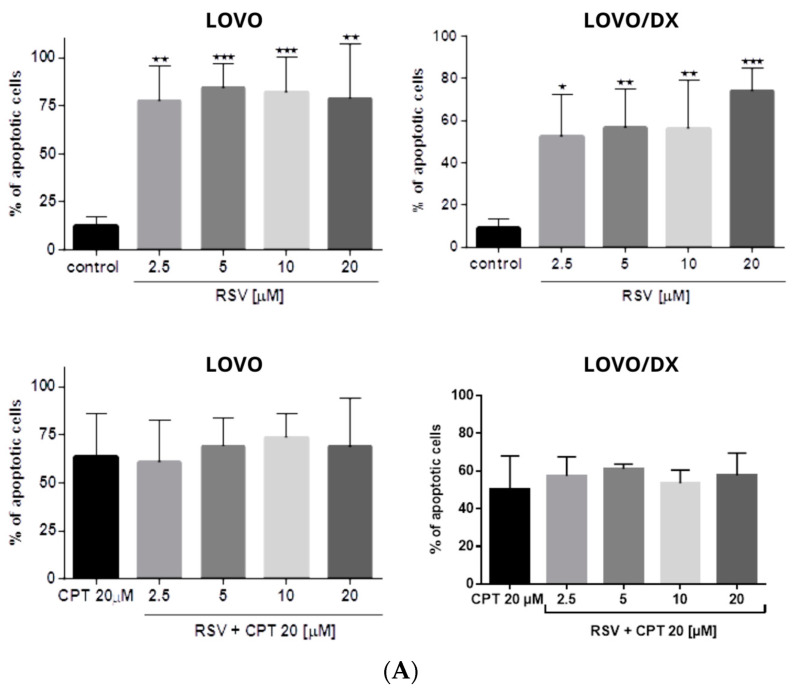

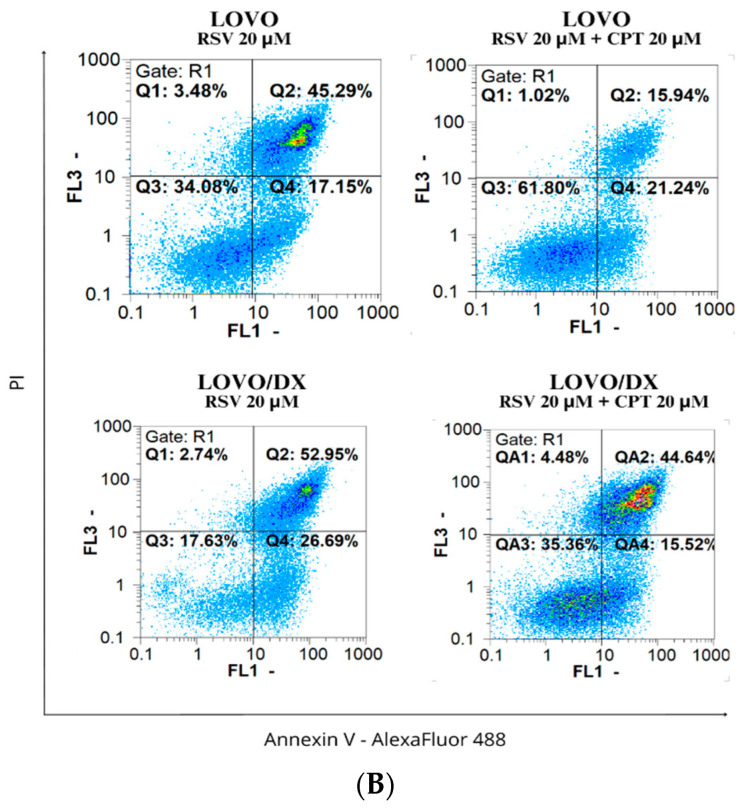
(**A**) Effects of resveratrol (RSV) and its combinations with camptothecin (CPT) on the frequency of apoptosis in LOVO and LOVO/DX cells. The results are presented as the mean ± SD of five independent experiments. * *p* ≤ 0.05, ** *p* < 0.01, *** *p* < 0.001. (**B**) Representative cytograms; FL1- Alexa Fluor^®^488 AnnexinV, FL3-Propidium Iodide (PI).

As shown in Figure 8, camptothecin strongly induces apoptosis of LOVO and LOVO/DX cells, with a greater effect on LOVO cells. The frequency of apoptotic cells increased up to 70% in LOVO cells and 50% in LOVO/DX cells. Celastrol and resveratrol show a strong pro-apoptotic effect in both cell lines; the number of cells showing features of apoptosis increased to 59% (CEL) and 84% (RSV) in LOVO cells and 66% (CEL) and 74% (RSV) in LOVO/DX cells (Figure 9 and Figure 10).

**Figure 11:**
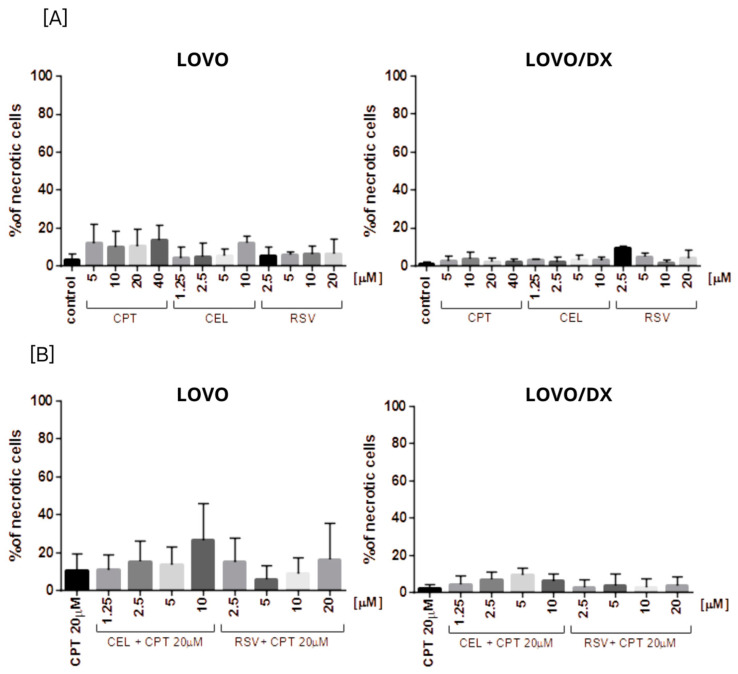
The effect of (**A**) camptothecin (CPT0, celastrol (CEL), resveratrol (RSV)) and (**B**) combinations of celastrol or resveratrol with camptothecin on the frequency of necrosis in LOVO and LOVO/DX cells. The results are presented as the mean ± SD of five independent experiments. *p* ≤ 0.05.

**Figure 12:**
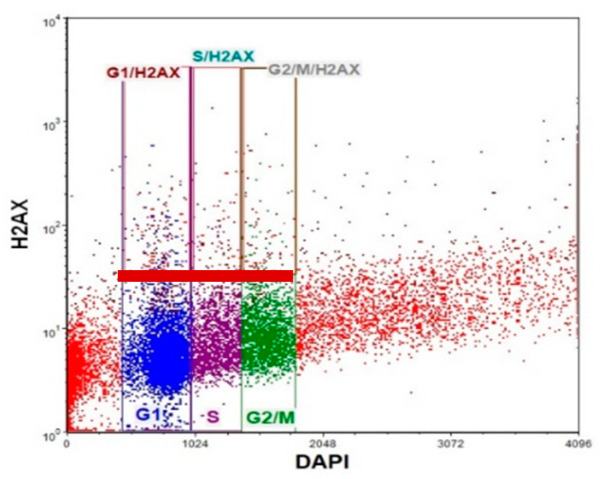
Representative cytogram of flow cytometric analysis of γH2AX in different phases of the cell cycle. Blue dots—G1 phase, purple dots—S phase, green dots—G2/M phase. Dots in red rectangle gates named G1/H2AX, S/H2AX, and G2/M/H2AX (over the red baseline) represent the γH2AX+cells in the corresponding cell cycle phase.

The combination of CPT (20 µM) with CEL or RSV does not enhance the pro-apoptotic effect of CPT alone, in both LOVO and LOVO/DX cells (Figure 11 and Figure 12). In LOVO cells, the combination of CPT-CEL decreased the frequency of apoptotic cells; nevertheless, the effect was not statistically significant. For other tested combinations, the effects were comparable to the effects of CPT alone. The calculated combination index was CI > 1 for all tested combinations.

None of the tested compounds and their combination induce significant cell necrosis (Figure 11). Very slight necrosis was noted only in LOVO cells for CEL (at 10 µM) and all concentrations of CPT. CPT increased the number of necrotic cells in the LOVO cell line by up to 15% and CPT-CEL (10 µM) by up to 20%.

### 3.3. Frequency of γ-H2AX in Different Phases of the Cell Cycle after Cell Exposure to Tested Compounds and Their Combination

It was demonstrated that DNA DSB-inducing agents cause an increase in γH2AX frequency during the progression from the G1 to S phase of the cell cycle. Thus, we next evaluated the level of γH2AX+ positive cells in different phases of the cell cycle in LOVO and LOVO/DX cells. We used double staining with anti-phosphohistone γH2AX (Ser139) Alexa Fluor 488 and DAPI DNA dye, followed by flow cytometric analysis. A representative cytogram with gating is shown in Figure 12.

**Figure 13:**
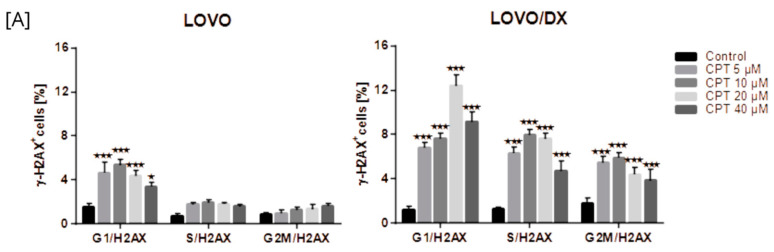

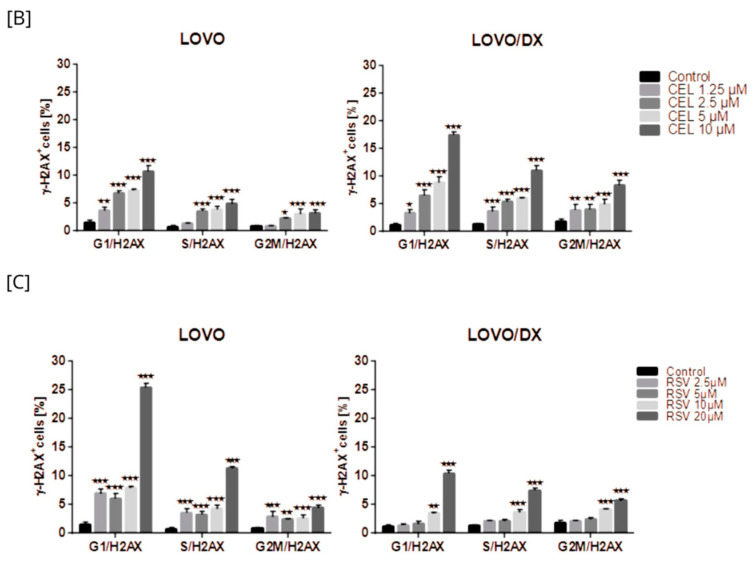
The effect of camptothecin (CPT) (**A**), celastrol (CEL) (**B**), or resveratrol (RSV) (**C**) on the frequency of γ-H2AX cells in different phases of the cell cycle, in LOVO and LOVO/DX cells. The results are presented as the mean ± SD of three independent experiments. * *p* ≤ 0.05, ** *p* < 0.01, *** *p* < 0.001.

**Figure 14:**
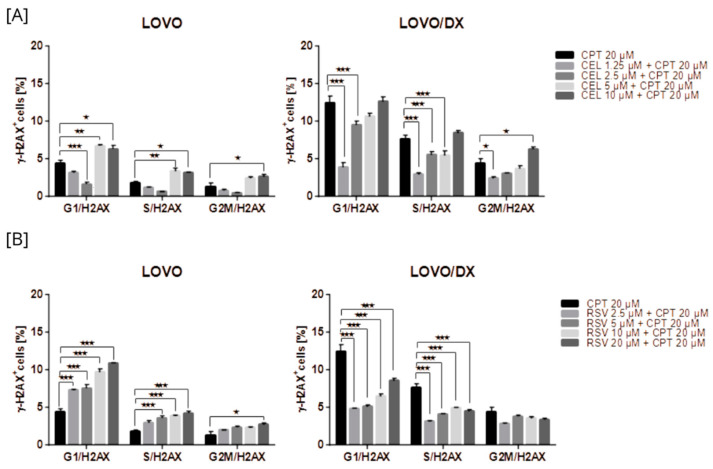
The effect of the combination of camptothecin (CPT) with celastrol (CEL) (**A**) or resveratrol (RSV) (**B**) on the frequency of γ-H2AX in different phases of the cell cycle, in LOVO and LOVO/DX cells. The results are presented as the mean ± SD of three independent experiments. * *p* ≤ 0.05, ** *p* < 0.01, *** *p* < 0.001.

Camptothecin raises the incidence of γH2AX+ positive cells in all phases of the cell cycle; however, the highest increase was noted for the G1 phase in LOVO cells and in the G1 and S phases in LOVO/DX cells (Figure 13A and Figure S7B). Similarly, exposure of LOVO and LOVO/DX cells to celastrol (Figure 13B and Figure S8SA) or resveratrol (Figure 13C and Figure S9A) more strongly increased the level of γH2AX+ positive cells in the G1 and S phases than in the G2M phase, especially at the highest concentrations. After LOVO and LOVO/DX cell exposure to the combination of CPT–CEL or CPT-RSV, the same general tendency was observed (Figure 14A,B, and Figures S8B and S9B). CPT-CEL treatment resulted in an increase in the number of γH2AX+ positive cells above the effect with CPT alone, at higher CEL concentrations, in all phases of the cell cycle. Whereas, CPT-RSV treatment induced an increase in γH2AX+ positive cells over the effect of CPT alone only in LOVO cells.

### 3.4. Effects of Camptothecin, Celastrol, Resveratrol, and Their Combination on Cancer Cells Migration

Cancer cells possess migration and invasion capacities. We further studied whether tested compounds and their combination inhibit cancer cell migration using the scratch test. The inhibition of cell migration was assessed by measuring the scratch closure over 72 h of incubation with the tested compound or their combination. Scratch closure under optimal conditions of LOVO and LOVO/DX cell culture is shown in Figure S10.

**Figure 15:**
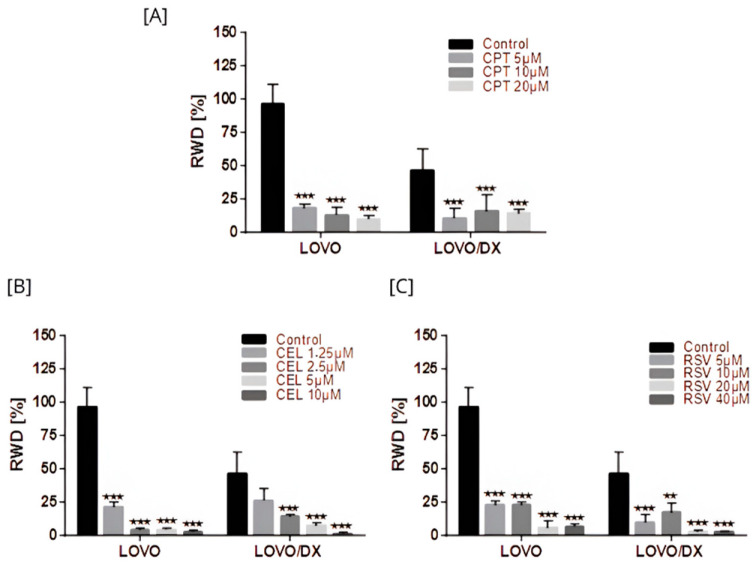
Effects of camptothecin (CPT) (**A**), celastrol (CEL) (**B**), and resveratrol (RSV) (**C**) on cancer cell migration. The results are presented as RWD (relative wound density) and are the mean ± SD of three independent experiments. ** *p* < 0.01, *** *p* < 0.001.

Camptothecin, celastrol, and resveratrol induced a strong inhibition of LOVO and LOVO/DX cell migration (Figure 15 and Figure S11). The strongest reduction in migration, over 90% in both cell lines, was noted for celastrol at 5 µM and 10 µM, and for resveratrol at 20 µM and 40 µM. This inhibitory effect was greater than that achieved by CPT.

**Figure 16:**
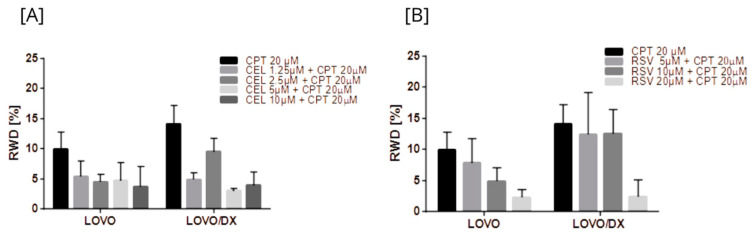
Effects of combinations of camptothecin (CPT) with celastrol (CEL) (**A**) or resveratrol (RSV) (**B**) on cancer cell migration. The results are presented as RWD (relative wound density) and are the mean ± SD of three independent experiments. *p* ≤ 0.05.

All combinations of CPT with CEL or RSV enhanced the effect of CPT alone (Figure 16 and Figure S12). Moreover, some combinations show a better effect than celastrol or resveratrol alone, i.e., CPT 20 µM + CEL 1.25 µM; CPT 20 µM + RSV 5 µM, 10 µM, 20 µM, 40 µM in LOVO cells, and CPT 20 µM + CEL 1.25 µM, 2.5 µM, 5 µM; and CPT 20 µM + RSV 5 µM, 10 µM in LOVO/DX cells. The calculated combination index was CI < 1 for all tested combinations in LOVO cells indicating a synergistic effect, while in LOVO/DX cells CI > 1.

## 4. Discussion

The standard chemotherapy for metastatic colorectal cancer is still based on a combination of three cytotoxic drugs: oxaliplatin (OXA) or irinotecan (IRY) with fluoropyrimidine, usually in double therapy [5]. The combination chemotherapy is, however, very intensive therapy and is associated with high general toxicity [4,5,6,7,8]. In addition, cytotoxic drugs usually target one survival pathway of cancer cells and eliminate rapidly proliferating cells without affecting drug-resistant cells, i.e., cancer stem cells [31]. Compounds of natural origin seem to have better anticancer properties compared to standard cytostatics because they have pleiotropic effects and are capable of interfering with various intracellular pathways. Furthermore, natural compounds are usually low-toxic, safe for healthy cells, and do not cause adverse reactions. However, some naturally occurring substances are not completely free of side effects or even exhibit significant toxicity [11].

An interesting approach is to combine phytochemicals with standard cytostatic drugs to improve their therapeutic effects on cancer cells and to protect healthy cells from the aggressiveness of chemotherapeutic agents [32,33]. For example, epigallocatechin-3-gallate (EGCG) in combination with doxorubicin exhibits a synergistic activity in prostate cancer cell proliferation and induces liver cancer cell death [34]. In CRC cells, the combination of 5-fluorouracil (5-FU) with EGCG, curcumin, or resveratrol increases the sensitivity to 5-FU cytotoxicity; the combination of alantolactone (sesquiterpene lactone) with OXA enhances apoptosis or EGCG combined with IRY causes stronger DNA damage, apoptosis, and suppression of cell migration [34,35,36,37,38,39].

In this study, we focused on the co-treatment of camptothecin with celastrol or resveratrol as potential strategies to target metastatic colon cancer cells (LOVO) and colon cancer cells enriched in cancer stem-like cells (LOVO/DX). Camptothecin was chosen as the representative compound of all CPT derivatives used in the clinic, including IRY, which is mostly used for the treatment of metastatic colon cancer. Celastrol and resveratrol are natural compounds known for their various biological effects and the ability to modify several key intracellular proteins, including p53, NF-κB, and Bax/Bcl2 [15,40]. We have previously demonstrated that both compounds can affect CRC cells through induction of DSB, apoptosis, and cell cycle arrest [21,23]. In addition, celastrol has been shown to have chemopreventive activity via inhibition of P-gp [22].

Camptothecin is a topoisomerase inhibitor by binding to topoisomerase I (TOP 1) and the DNA complex, which prevents DNA re-ligation and thus causes DNA damage [41,42]. Genomic instability resulting from DNA damage can ultimately lead to cancer development. It is worth noting that most anticancer drugs, such as cisplatin, bind to DNA, altering its structure and causing DNA damage. Furthermore, this DNA damage can lead to cell cycle inhibition and induce apoptosis in rapidly proliferating cancer cells [43,44]. For studying the genotoxicity of camptothecin and its co-treatment with CEL or RSV in LOVO and LOVO/DX cells, we used the γH2AX+ assay and the FHA assay. Both assays allow us to evaluate early and late genotoxic effects and to estimate the occurrence of double DNA damages, which are lethal to the cell because they involve double strands of DNA and result in the loss of genetic material [28,45,46]. Camptothecin, in monotherapy, induces γH2AX formation and DNA fragmentation, indicating the presence of DNA damage. However, the sensitivity of LOVO and LOVO/DX cells to camptothecin was moderate. It was previously demonstrated by Roy A. and Tesauro C. groups that CD44+ CSCs were resistant toward camptothecin treatment and this resistance was associated with reduced activity of the TOP1 in those cells [47,48]. Our results are consistent with these observations, as both LOVO and LOVO/DX cells express the CD44 antigen, which may explain the lower genotoxic effect of camptothecin in these cells [21]. On the other hand, celastrol and resveratrol have stronger genotoxic effects, which could potentially improve the effects of camptothecin. Celastrol significantly improves the effect of camptothecin, especially in CSC enriched LOVO/DX cells, increasing both early and late genotoxic effects (as measured by γH2AX+ formation and DNA fragmentation). In addition, all combinations of CPT with CEL demonstrated synergistic effects in LOVO/DX cells. Metastatic cells (LOVO cells) appear to be less sensitive to CPT-CEL co-treatment, and only late genotoxic effects could be detected (much weaker than in LOVO/DX cells). Moreover, lower levels of γH2AX+ were observed in metastatic cells after treatment with CPT-CEL compared to CEL and CPT alone, showing antagonistic effects. A possible explanation for this phenomenon is that metastatic (drug-sensitive) cells undergo DNA damage more quickly and that γH2AX in the presence of CPT-CEL is less detectable. This correlates with the reported increased levels of DNA fragmentation compared to celastrol or camptothecin alone. Camptothecin is a P-gp substrate and effluxes by this transporter reducing its intracellular concentration [49]. LOVO/DX cells are characterized by enhanced expression of MDR-associated transporters, including P-gp [21]. Moreover, celastrol can inhibit the transport function of P-gp [22]. It explains the better effects of the CPT-CEL combination in LOVO/DX cells. In contrast, resveratrol alone induces stronger DSBs in LOVO cells and improves the efficiency of camptothecin in those cells, as detected by increased levels of phosphorylated H2AX and DNA fragmentation after CPT-RSV treatment relative to CPT. However, the calculated combination index (CI) indicates antagonistic effects of camptothecin and resveratrol in LOVO cells. This is due to the strong genotoxic effects of RSV alone and weaker effects of CPT-RSV treatment compared to RSV. In CSCs, RSV and the combination of CPT with RSV only increase the degree of DNA fragmentation, and the effect is stronger than in LOVO cells. Calculated CI indicates a synergistic effect of camptothecin and resveratrol in those cells. However, it appears that CPT-RSV treatment induces only a slight increase in DNA damage compared to RSV alone, which may be related to its strong antioxidant properties that probably overlap with upregulated antioxidant systems in LOVO/DX cells. As demonstrated by Demoulin B. et al., RSV-induced DNA damage appears to rely on type II topoisomerase poisoning rather than on the pro-oxidant effect of the molecule, inhibition of topoisomerase I, or DNA intercalation [50]. IT is worth noting that γH2AX is considered a highly sensitive DSB marker; however, it does not directly measure DSBs. The FHA test detects the degree of DNA breakage caused by different types of DNA damage that lead to DNA fragmentation [45]. This suggests that other than DSB mechanisms are also involved in celastrol and resveratrol-induced genotoxic effects against metastatic LOVO cells enriched in CSC LOVO/DX cells.

Apoptosis is a secondary response to DNA damage and an important mechanism of tumor suppression in cancer chemotherapy [51]. In our study, camptothecin induces strong apoptosis and slight necrosis in LOVO cells and has important proapoptotic effects on LOVO/DX cells. However, these results do not correlate with the reported significantly lower genotoxic effect of camptothecin, indicating that the induction of apoptosis by camptothecin might be governed by a different pathway in metastatic LOVO cells and enriched in CSCs LOVO/DX cells. For example, Han Z. et al. demonstrated that camptothecin-induced apoptosis occurred through a p53 and p21-independent mechanism [52]. Mitochondrial fission has also been recently reported to be an important process in the induction of apoptosis by camptothecin [53]. Furthermore, Park K. et al. showed that hypoxia attenuates camptothecin-induced apoptosis by reducing Bax protein levels, thereby leading to resistance to the drug [54]. Celastrol and resveratrol show strong proapoptotic effects in both LOVO and LOVO/DX cells. According to the genotoxic results, celastrol induces a slightly higher level of apoptosis in enriched in CSCs LOVO/DX cells whereas resveratrol applies in metastatic LOVO cells. Surprisingly, combining camptothecin with celastrol or resveratrol does not enhance the proapoptotic effect of camptothecin alone. It also does not increase cancer cell necrosis. Since emerging evidence suggests that different types of cell death often share common pathways, it can be hypothesized that the CPT-CEL or CPT-RSV treatment, in addition to inducing apoptosis, leads to other non-apoptotic cell deaths such as pyroptosis, ferroptosis, autophagy, or oncosis [55]. For example, celastrol has been reported to induce ferroptosis by promoting the production of reactive oxygen species (ROS) whereas resveratrol induces different types of autophagy [56,57]. The crosstalk between different types of cell death is complex and has wide implications for the fate of cancer cells. Apoptosis and autophagy share common upstream triggers, as well as autophagy-related proteins and apoptotic proteins. A mutual promotion between apoptosis and lethal autophagy has been observed. However, inhibition of autophagy has also been reported to significantly promote apoptosis, indicating that autophagy has an antagonistic effect on apoptosis [57]. The possibility that a combination treatment of camptothecin with celastrol or resveratrol could switch between different types of cell death should be explored.

MacPhail et al. demonstrated that DNA DSB-inducing agents cause an increase in γH2AX levels during the progression from the G1 to S phase of the cell cycle [58]. Here, we showed that the incidence of γH2AX+ positive cells increased in all phases of the cell cycle after treatment with camptothecin alone and CPT-CEL or CPT-RSV combinations in both LOVO and LOVO/DX. However, a higher amount of γH2AX+ cells was detected in G1 and S phases. The S phase is a very important stage of cell cycle progression, as it allows for proper replication of DNA [23]. Moreover, the G2/M DNA damage checkpoint serves to prevent the cell from entering mitosis with genomic DNA damage [59]. An increase in DSB in the early stages of cell cycle progression is usually associated with cell cycle arrest, inhibition of cell proliferation, and initiation of processes such as cell death (including apoptosis) or senescence [60]. Several DSB-inducing anticancer drugs cause cell-cycle arrest associated with the inhibition of cell proliferation [23]. We previously showed that celastrol and resveratrol induce cell cycle arrest in the S stage in both metastatic and enriched in CSC colon cancer cell lines [21]. Yoo J-m et al. reported that camptothecin induces G1 phase arrest by blocking the DNA topoisomerase complex [61].

Cancer cell migration is a key stage in solid tumor metastasis and invasion. Li B-S et al. reported that camptothecin treatment inhibits the migration and invasion of nasopharyngeal carcinoma cells [62]. Another study demonstrated that camptothecin abrogates cell motility and the invasion of HCC cells by modulating the expression of miRNAs [63]. Consistent with previous studies, we observed that camptothecin, in LOVO cells and LOVO/DX cells, induces strong inhibition of cell migration. A comparable effect was noted for celastrol and resveratrol, according to the literature reports [64,65]. Importantly, the combination of CPT-CEL or CPT-RSV enhances the anti-migratory effect of camptothecin alone. Recently, Piet M. et al. showed that ursolic acid (UA) and oleanolic acid (OA), plant-derived pentacyclic triterpenoids, when combined with irinotecan, inhibit migration of the cancer cells [66].

## 5. Conclusions

We showed that metastatic LOVO cells and cells enriched in CSC LOVO/DX are sensitive to the genotoxic activity of camptothecin, celastrol, and resveratrol. The combination of natural compounds, i.e., celastrol or resveratrol with camptothecin, improves the general genotoxicity of camptothecin alone. However, measuring specific DSBs by sensitive γH2AX+ assay indicates a stronger effect of the combination of camptothecin with celastrol in enriched in CSC LOVO/DX cells and camptothecin with resveratrol in metastatic LOVO cells. Interestingly, none of the tested combinations improve the proapoptotic effect of camptothecin, suggesting that other consequences of genotoxic effects might be involved, i.e., activation of other non-apoptotic cell deaths and inhibition of proliferation. Moreover, we found that the combination of camptothecin with celastrol or resveratrol improves the inhibitory effect of camptothecin alone on cell migration. Our findings suggest the potential usefulness of such double therapy in the treatment of more aggressive forms of colon cancer. Due to the natural origin of celastrol and resveratrol, potential clinical implications in the treatment of colorectal cancer may be considered in the future. In addition, it might be suggested that the combination of celastrol with camptothecin (or its derivatives) could be the best treatment for eliminating CSCs, whereas the combination of resveratrol with camptothecin (or its derivatives) will be more effective in the case of metastatic cells. It is worth noting that the combination of celastrol and resveratrol with camptothecin has not been tested so far, and no studies evaluating the effects of these compound combinations have been found in the available medical databases.

## Figures and Tables

**Table 1:** Combination index (CI) values were obtained for camptothecin (CPT) and celastrol (CEL) combinations based on the genotoxic effect. The Chou–Talalay method was used and the values were calculated using the CompuSyn software. The combination index defines synergism when CI < 1, additive effect when CI = 1, and antagonism effects when CI > 1.

	CI (Combination Index)	
	LOVO	LOVO/DX
CPT 20 µM + CEL 1.25 µM	1.2	0.3
CPT 20 µM + CEL 2.5 µM	2.8	0.4
CPT 20 µM + CEL 5 µM	3.0	0.5
CPT 20 µM + CEL 10 µM	>10	0.6

**Table 2:** Combination index (CI) values were obtained for camptothecin (CPT) and resveratrol (RSV) combinations based on the genotoxic effect. The Chou–Talalay method was used and the values were calculated using the CompuSyn software. The combination index defines synergism when CI < 1, additive effect when CI = 1, and antagonism effects when CI > 1.

	CI (Combination Index)	
	LOVO	LOVO/DX
CPT 20 µM + RSV 2.5 µM	8.4	0.5
CPT 20 µM + RSV 5 µM	>10	0.6
CPT 20 µM + RSV 10 µM	>10	0.6
CPT 20 µM + RSV 20 µM	>10	1.1

## Data Availability

The data that support the findings of this study are openly available at the request of the reader.

## Supplementary Materials

The following supporting information can be downloaded at: https://www.mdpi.com/article/10.3390/cancers16193279/s1, Table S1: Combination index values and descriptions for classifying synergism or antagonism using the Chou-Talalay method (from Matthews H. et al. [31]); Figure S1: Dose-effect curve for camptothecin (5 µM, 10 µM, 20 µM, 40 µM), celastrol (1.25 µM, 2.5 µM, 5 µM, 10 µM), and combinations of CPT (20 µM) with CEL at 1.25 µM, 2.5 µM, 5 µM or 10 µM; Figure S2: Dose-effect curve for camptothecin (5 µM, 10 µM, 20 µM, 40 µM), resveratrol (2.5 µM, 5 µM, 10 µM, 20 µM), and combinations of CPT (20 µM) with RSV at 2.5 µM, 5 µM, 10 µM or 20 µM; Figure S3: Screenshots of the “Halo Assay” software (by Benita Wiatrak) showing the determination of the ratio of the cell nucleus diameter to the diameter of the HALO glow; Figure S4: Dose-effect analysis for camptothecin (20 µM), celastrol (1.25 µM, 2.5 µM, 5 µM, 10 µM), and combinations of CPT (20 µM) with CEL at 1.25 µM, 2.5 µM, 5 µM or 10 µM; Figure S5: Dose-effect analysis for camptothecin (20 µM), resveratrol (5 µM, 20 µM), and combinations of CPT (20 µM) with RSV at 5 µM or 20 µM; Figure S6: Representative cytograms of flow cytometric analysis of apoptosis in LOVO and LOVO/DX control cells; Figure S7: Frequency of γ-H2AX in different phases of the cell cycle in control cells (A) and after cell exposure to camptothecin (CPT) (B), in LOVO and LOVO/DX. The results are shown as representative cytograms; Figure S8: Frequency of γ-H2AX in different phases of the cell cycle after cell exposure to celastrol (A) and its combinations with camptothecin (B), in LOVO and LOVO/DX cells; Figure S9: Frequency of γ-H2AX in different phases of the cell cycle after cell exposure to resveratrol (A) and its combinations with camptothecin (B), in LOVO and LOVO/DX cells; Figure S10: Representative photos of scratch closure after 72 h of incubation of LOVO and LOVO/DX cells under optimal conditions; Figure S11: Representative photos of scratch closure after incubation of LOVO and LOVO/DX cells with (A) camptothecin (CPT), (B) celastrol (CEL), (C) resveratrol (RSV); Figure S12: Representative photos of scratch closure after LOVO and LOVO/DX cells incubation (72 h) with the combination of camptothecin (CPT) with celastrol (CEL) (A) or resveratrol (RSV) (B).
