# Supplementary material for: Genotoxic and Anti-Migratory Effects of Camptothecin Combined with Celastrol or Resveratrol in Metastatic and Stem-like Cells of Colon Cancer
Source: Cancers (Basel). 2024 Sep 26;16(19):3279. doi: 10.3390/cancers16193279 (PMC11476312; doi:10.3390/cancers16193279)
Supplement: Supplementary file 1 [file cancers-16-03279-s001.zip › cancers-3198678-supplementary.pdf]

Supplementary Figures and Tables

Combination Index (CI) equation of Chou-Talalay for two drugs :

$$CI = (D)_1/(D_x)_1 + (D)_2/(D_x)_2,$$

where $(D_x)_1$ is the doses of drug 1 alone that inhibits x%, $(D_x)_2$ is the doses of drug 2 alone that inhibits x%, $(D)_1$ is the portion of drug 1 in combination $(D)_1 + (D)_2$ inhibits x% and $(D)_2$ is the portion of drug 1 in combination $(D)_1 + (D)_2$ inhibits x%.

From:
„CompuSyn for Drug Combinations and for General Dose-Effect Analysis User's Guide. A Computer Program for Quantitation of Synergism and Antagonism in Drug Combinations, and the Determination of IC50, ED50, and LD50 Values" by Ting-Chao Chou and Nick Martin Published and Distributed by ComboSyn, Inc. Copyright 2005, https://www.combosyn.com/

Table S1. Combination index values and descriptions for classifying synergism or antagonism using the Chou-Talalay method (from Matthews H. et al. [31]).

Range of CI	Description
<0.1	Very strong synergism
0.1±0.3	Strong synergism
0.3±0.7	Synergism
0.7±0.85	Moderate synergism
0.85±0.90	Slight synergism
0.90±1.10	Nearly additive
1.10±1.20	Slight antagonism
1.20±1.45	Moderate antagonism
1.45±3.3	Antagonism
3.3±10	Strong antagonism
>10	Very strong antagonism

LOVO LOVO/DX

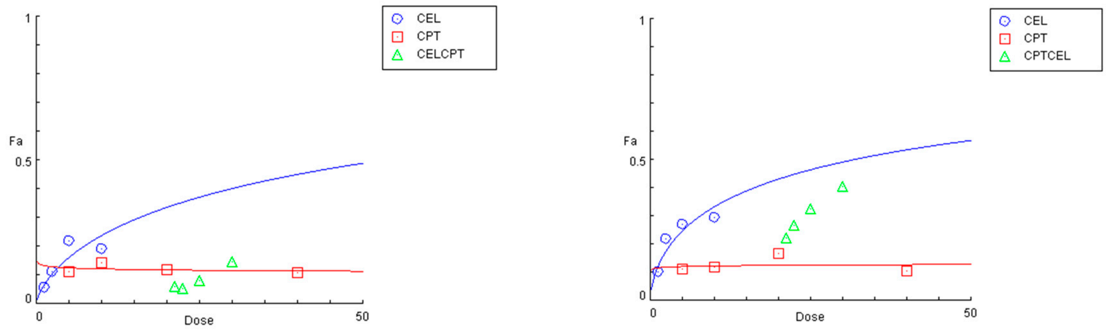

Figure S1. Dose-effect curve for camptothecin (5µM, 10µM, 20µM, 40µM), celastrol (1.25µM, 2.5µM, 5µM, 10µM), and combinations of CPT (20µM) with CEL at 1.25µM, 2.5µM, 5µM or 10µM. Dose-effect analysis was completed using CompuSyn software (ComboSyn, Inc., Paramus, NJ. 07652 USA).

LOVO LOVO/DX

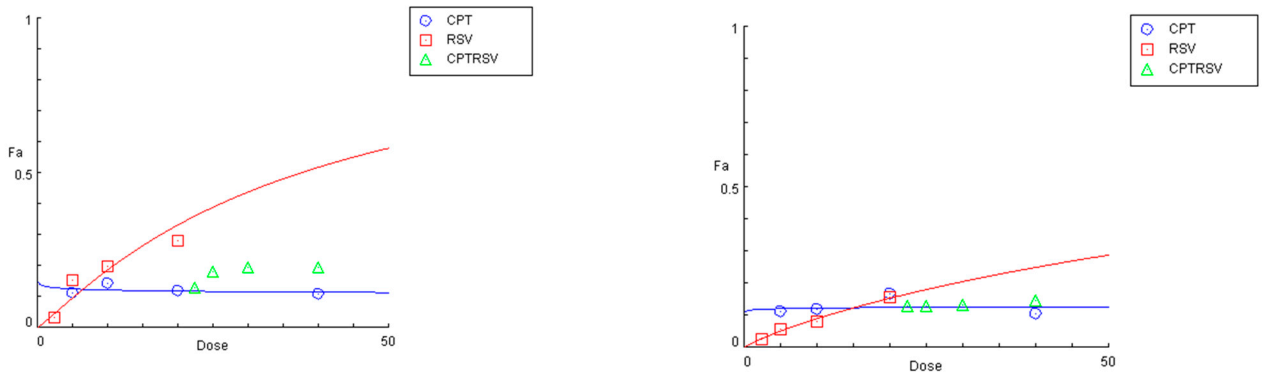

Figure S2. Dose-effect curve for camptothecin (5µM, 10µM, 20µM, 40µM), resveratrol (2.5µM, 5µM, 10µM, 20µM), and combinations of CPT (20µM) with RSV at 2.5µM, 5µM, 10µM or 20µM. Dose-effect analysis was completed using CompuSyn software (ComboSyn, Inc., Paramus, NJ. 07652 USA).

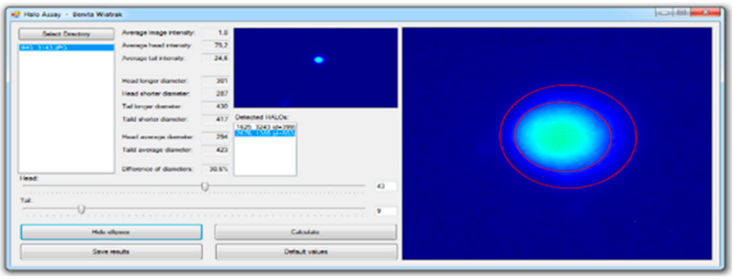

Figure S3. Screenshots of the "Halo Assay" software (by Benita Wiatrak) showing the determination of the ratio of the cell nucleus diameter to the diameter of the HALO glow.

LOVO LOVO/DX

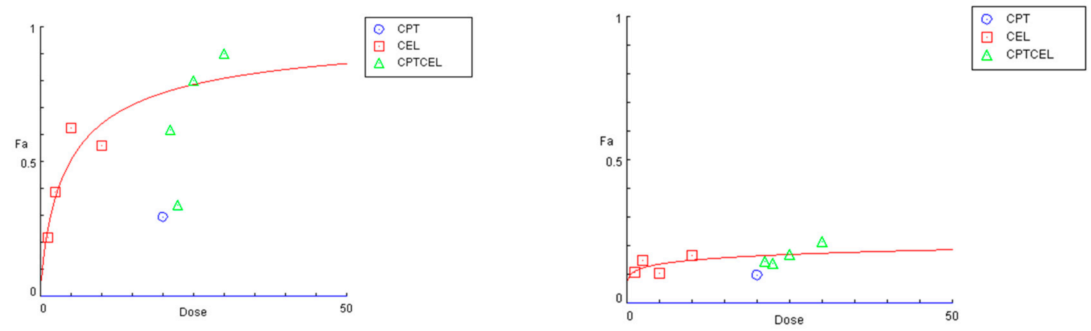

Figure S4. Dose-effect analysis for camptothecin (20μM), celastrol (1.25μM, 2.5μM, 5μM, 10μM), and combinations of CPT (20μM) with CEL at 1.25μM, 2.5μM, 5μM or 10μM. The analysis was completed using CompuSyn software (ComboSyn, Inc., Paramus, NJ. 07652 USA).

LOVO LOVO/DX

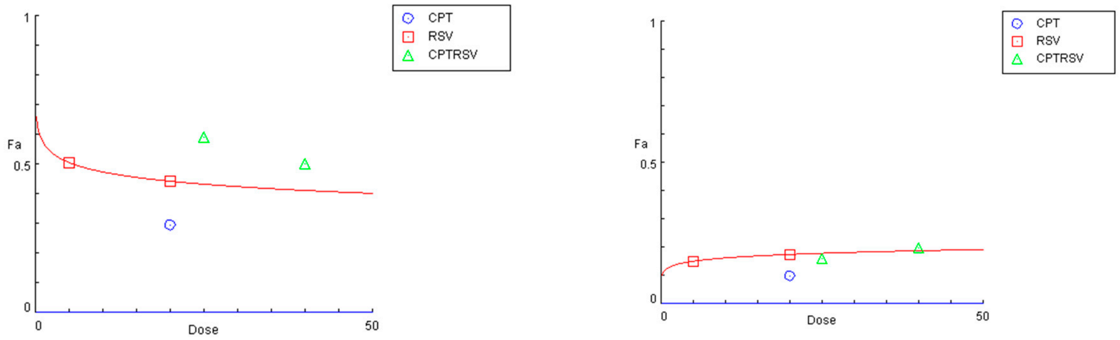

Figure S5. Dose-effect analysis for camptothecin (20μM), resveratrol (5μM, 20μM), and combinations of CPT (20μM) with RSV at 5μM or 20μM. The analysis was completed using CompuSyn software (ComboSyn, Inc., Paramus, NJ. 07652 USA).

LOVO CONTROL **LOVO/DX CONTROL**

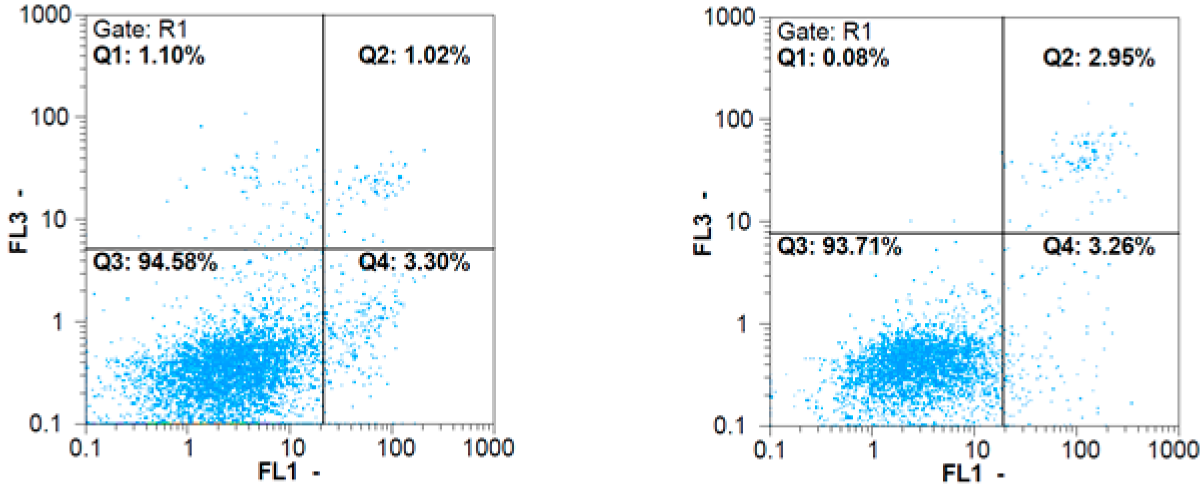

Figure S6. Representative cytograms of flow cytometric analysis of apoptosis in LOVO and LOVO/DX control cells. FL1- Alexa Fluor®488 AnnexinV, FL3- Propidium Iodide (PI).

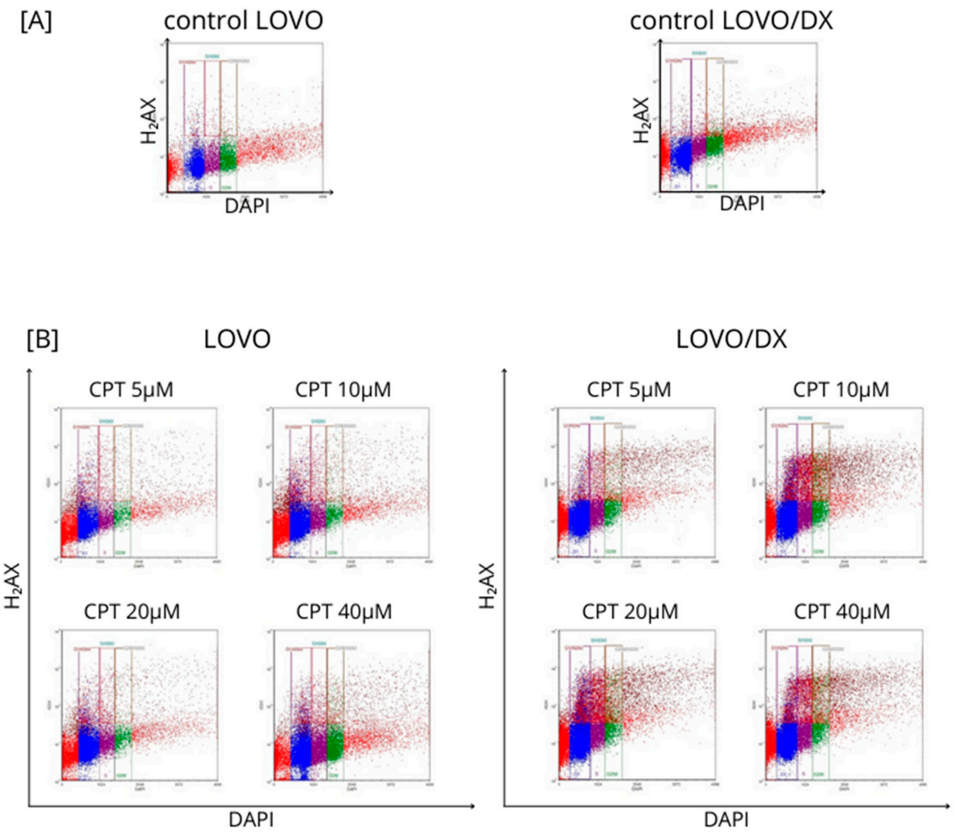

Figure S7. Frequency of γ-H2AX in different phases of the cell cycle in control cells [A] and after cell exposure to camptothecin (CPT) [B], in LOVO and LOVO/DX. The results are shown as representative cytograms.

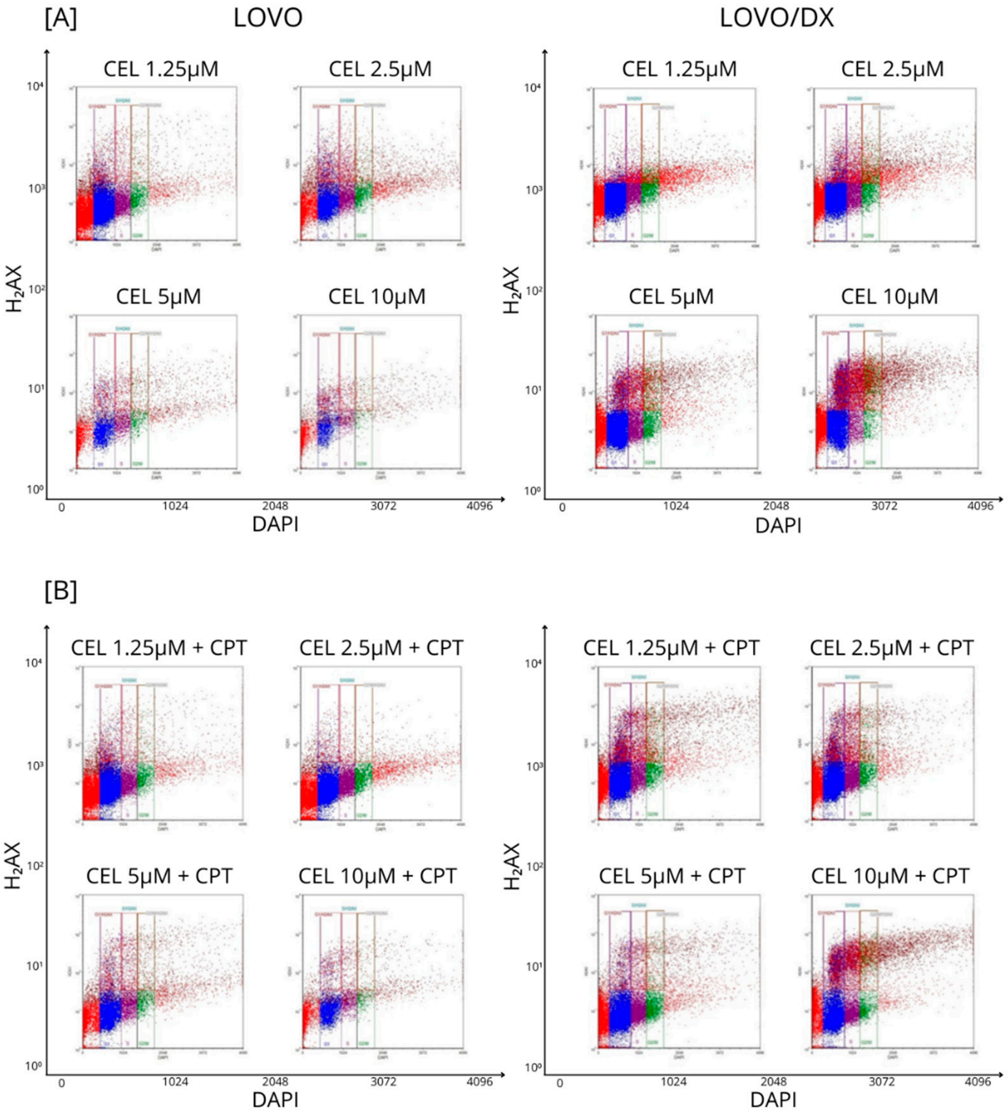

Figure S8. Frequency of γ-H2AX in different phases of the cell cycle after cell exposure to celastrol [A] and its combinations with camptothecin [B], in LOVO and LOVO/DX cells. The results are shown as representative cytograms.

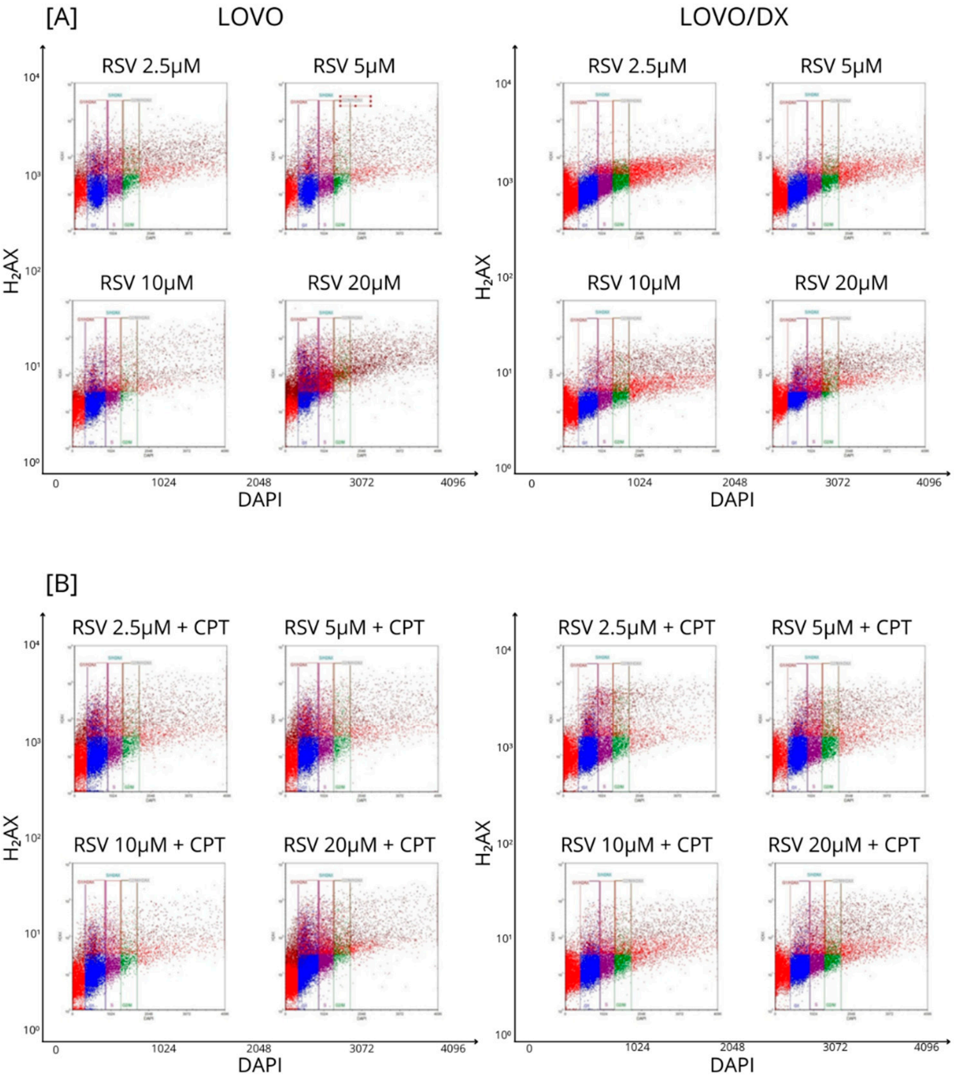

Figure S9. Frequency of γ-H2AX in different phases of the cell cycle after cell exposure to resveratrol [A] and its combinations with camptothecin [B], in LOVO and LOVO/DX cells. The results are shown as representative cytograms.

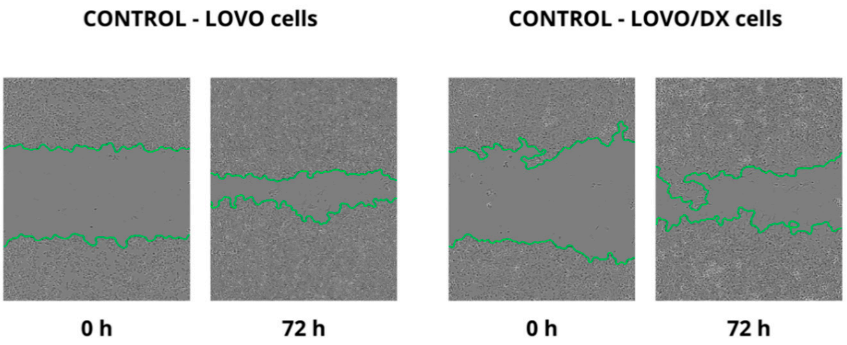

Figure S10. Representative photos of scratch closure after 72 hours of incubation of LOVO and LOVO/DX cells under optimal conditions.

[A]

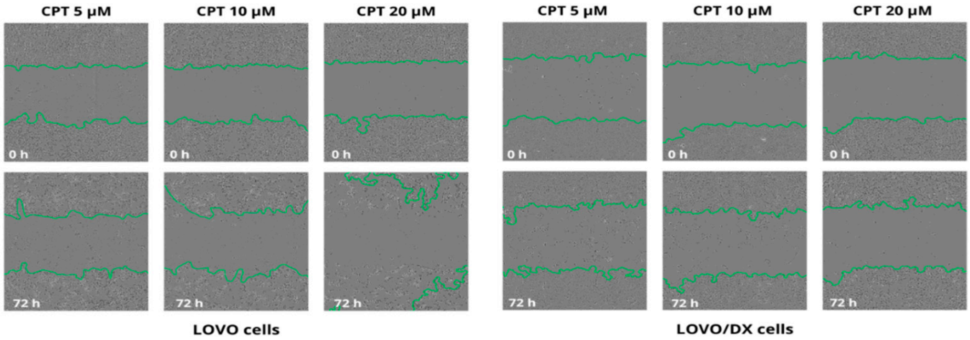

[B]

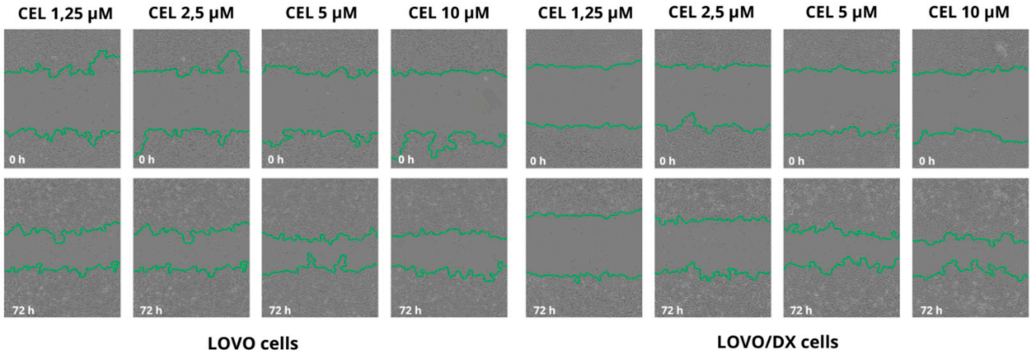

[C]

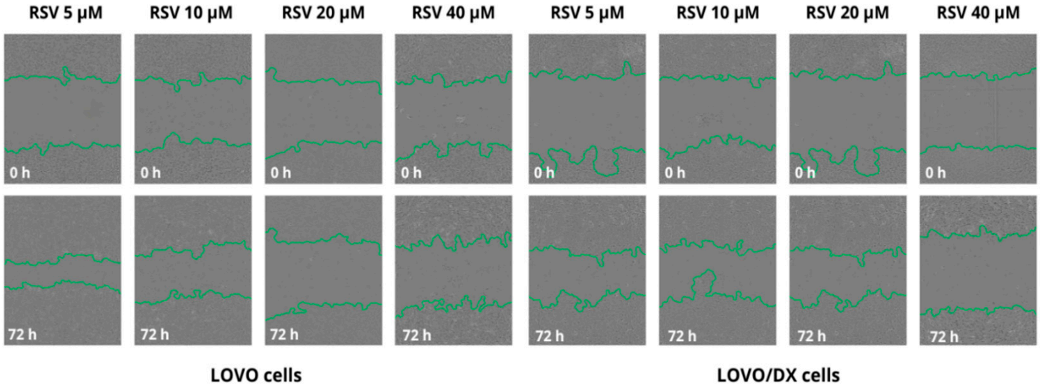

Figure S11. Representative photos of scratch closure after incubation of LOVO and LOVO/DX cells with [A] camptothecin (CPT), [B] celastrol (CEL), [C] resveratrol (RSV).

[A]

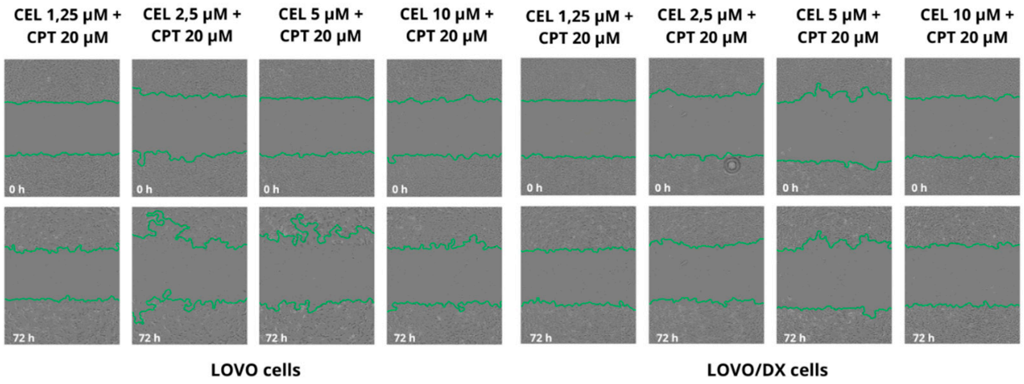

[B]

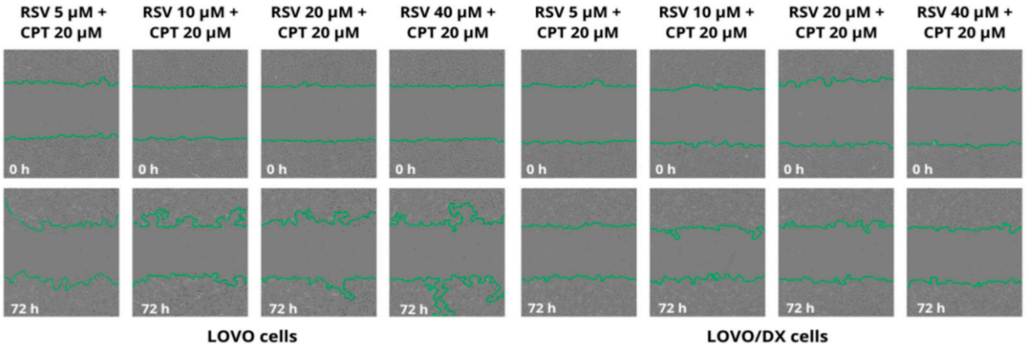

Figure S12. Representative photos of scratch closure after LOVO and LOVO/DX cells incubation (72 hours) with the combination of camptothecin (CPT) with celastrol (CEL) [A] or resveratrol (RSV) [B].
